# On-field phenotypic evaluation of sunflower populations for broad-spectrum resistance to Verticillium leaf mottle and wilt

**DOI:** 10.1038/s41598-021-91034-4

**Published:** 2021-06-02

**Authors:** Juan F. Montecchia, Mónica I. Fass, Ignacio Cerrudo, Facundo J. Quiroz, Salvador Nicosia, Carla A. Maringolo, Julio Di Rienzo, Carolina Troglia, H. Esteban Hopp, Alberto Escande, Julio González, Daniel Álvarez, Ruth A. Heinz, Verónica V. Lia, Norma B. Paniego

**Affiliations:** 1 Instituto de Agrobiotecnología Y Biología Molecular (IABIMO), Instituto Nacional de Tecnología Agropecuaria (INTA), Consejo Nacional de Investigaciones Científicas Y Técnicas (CONICET), Hurlingham B1686IGC, Buenos Aires, Argentina; 2 Instituto Nacional de Tecnología Agropecuaria (INTA), Estación Experimental Agropecuaria Balcarce, Buenos Aires, Argentina; 3 Facultad de Ciencias Agropecuarias, Universidad Nacional de Córdoba, Córdoba, Argentina; 4 Facultad de Ciencias Exactas Y Naturales, Universidad de Buenos Aires, Buenos Aires, Argentina; 5 Instituto Nacional de Tecnología Agropecuaria (INTA), Estación Experimental Agropecuaria Pergamino, Buenos Aires, Argentina; 6 Instituto Nacional de Tecnología Agropecuaria (INTA), Estación Experimental Agropecuaria Manfredi, Manfredi, Córdoba, Argentina

**Keywords:** Agricultural genetics, Biotic, Plant breeding, Natural variation in plants

## Abstract

Sunflower Verticillium Wilt and Leaf Mottle (SVW), caused by *Verticillium dahliae* (Kleb.; *Vd*), is a soil-borne disease affecting sunflower worldwide. A single dominant locus, known as V1, was formerly effective in controlling North-American *Vd* races, whereas races from Argentina, Europe and an emerging race from USA overcome its resistance. This emphasizes the need for identifying broad-spectrum genetic resistance (BSR) sources. Here we characterize two sunflower mapping populations (MPs) for SVW resistance: a biparental MP and the association MP from the National Institute of Agricultural Technology (INTA), under field growing conditions. Nine field-trials (FTs) were conducted in highly infested fields in the most SVW-affected region of Argentina. Several disease descriptors (DDs), including incidence and severity, were scored across four phenological stages. Generalized linear models were fitted according to the nature of each variable, adjusting mean phenotypes for inbred lines across and within FTs. Comparison of these responses allowed the identification of novel BSR sources. Furthermore, we present the first report of SVW resistance heritability, with estimates ranging from 35 to 45% for DDs related to disease incidence and severity, respectively. This study constitutes the largest SVW resistance characterization reported to date in sunflower, identifying valuable genetic resources for BSR-breeding to cope with a pathogen of increasing importance worldwide.

## Introduction

Sunflower Verticillium Wilt and Leaf Mottle (SVW) is a monocyclic vascular disease whose causative agent is the soil-borne fungal pathogen *Verticillium dahliae* (Kleb.). Fungal inoculum consists of long-lasting microsclerotia, which remain infective in soil from 10 to 15 years^1^. *V. dahliae* (*Vd*) is a polyphagous pathogen affecting over 350 dicotyledonous hosts, thus making its management difficult through common agricultural practices^2–5^. The wilting caused by SVW exerts up to 30% yield reductions in susceptible commercial sunflower hybrids^6,7^ and up to 73% in susceptible materials grown in highly infested fields^8^. Yield losses have a direct relationship with symptom level (i.e. foliage necrotic surface)^7,9^.

SVW occurs in most sunflower producing areas of the world^10^. Historically, this has been the most prevalent disease in Argentina and it has high impact on extensive regions of Canada and USA, where new pathogenic races have arisen^1,5,11,12^. Moreover, it has recently become a serious threat for European sunflower producing countries of temperate regions with increasing prevalence rates in France, Italy, Spain and countries around the Black Sea^13,14^.

In Argentina, the third sunflower producer and edible-oil exporter worldwide^15,16^, *Vd* is an endemic pathogen with a variety of local races^17–19^. *Vd* inoculum is spread over 1.2 million hectares, affecting over 70% of the country’s sunflower growing region^20^. In the south of Buenos Aires Province, the main area for sunflower production in Argentina, the SVW-prevalence levels have shown an average of 45% (± 14%) over the last 8 years^21^.

Despite the relevance of the pathogen, the disease-management tools available to date are limited. Although no-tilling is known to be a useful tool for lowering disease incidence^4^, genetic resistance is still the most effective strategy to cope with this affection. SVW resistance was first reported as a qualitative trait governed by a single dominant locus^22^. Later contributions have described different inbred lines (ILs) with dominant, additive and recessive sources of resistance to North American races^23,24^. Since then, the V1 locus, which was identified on the maintainer IL HA89^25^, has been the main source of resistance for hybrid development worldwide^12^.

During the last 40 years, various studies have reported *Vd* races overcoming V1 locus resistance, first in Argentina and later in the USA and Europe^14,17,26,27^. In Argentina, two local races, VArg1 and VArg2, were identified among the isolates affecting sunflower^18^. Consequently, researchers of the private sector have reported pairs of differential inbred lines bearing specific resistance to these races, giving rise to SVW-resistant hybrids^7,18,28^. However, further reports described less-frequent new races overcoming these resistance sources as well^19^.

Even though some commercial hybrids currently display SVW-resistance, the lack of public research on this subject has prevented disentangling the genetic architecture of the trait. Exploring large sets of germplasm and characterizing their behavior against SVW are the first steps towards identifying novel resistance sources to contribute to sunflower breeding. However, no large-scale surveys have been conducted and little is known about the importance of the different DDs (i.e. disease incidence, disease severity, disease intensity) or their heritability, up to this date.

In this study, we evaluated 301 ILs of two mapping populations (MPs) developed by the INTA for SVW Broad Spectrum Resistance (BSR) against Argentinian local races. The first MP is the result of the biparental crossing between two restorer public-ILs PAC2 × RHA439 (biparental mapping panel, BMP). The second is the sunflower association mapping panel of INTA (AMP)^29–32^. The AMP was extended for this study by incorporating ILs from the INTA breeding program with good behavior for SVW-resistance formerly identified by breeders on the EEA-INTA-Pergamino using artificial inoculation^33^. The AMP contains a representative sample of the genetic diversity held in INTA’s sunflower breeding program and comprises 164 ILs. The genetic diversity of the AMP is comparable to that of other sunflower inbred panels from public breeding programs from France, Canada and USA, while containing the singularities of the Argentinian germplasm^32^.

The aims of the present study were: (1) to assess the diversity held in INTA´s genetic resources for SVW-resistance, (2) to identify inbred lines representing new SVW-resistance sources, (3) to understand the inheritance and (iv) to estimate the heritability of the trait.

## Results

### Raw data descriptive statistics across field trials

The phenotypic evaluation for SVW resistance of the two MPs retrieved robust results for 162 ILs from the AMP (5 field trials, in two locations) and 139 RILs from the BMP (4 FTs in one location). SVW was scored at four Evaluation Dates (EDs). The DDs recorded to evaluate ILs at each ED were: disease incidence (DI), disease severity (DS), binomial disease severity (bDS) and disease intensity (DInt). Estimation of the Area under the Disease Progress Curve for disease incidence (AUDPC.DI) and for disease intensity (AUPDC.DInt) integrated all EDs. The combination of DDs and EDs rendered 18 SVW-DDs to be modeled in the statistical analysis. We selected four of those to illustrate IL’s behavior on the basis of their potential impact on yield components. The selected combinations were DI at flowering (DI.Flw or DI.3), AUDPC.DI, DS at grain filling (DS.Gf or DS.4) and bDS at grain filling (bDS.Gf or bDS.4).

The analysis of the raw DI.Flw and DS.Gf values showed similar overall patterns in both MPs, with DI.Flw and DS.Gf ranging from 1.6 to 7.3 plants per plot (p.p.p.) and from 2.9 to 3.9 symptomatic p.p.p., respectively (Table 1).

**Table 1:** Across field trial means and ranges, in brackets, for raw measurements of number of plants per plot (p.p.p.), symptomatic p.p.p. at flowering, average DS.Gf score symptomatic plants and overall proportion of DI.Flw across field trials.

Mapping panel	Average number of p.p.p	Average symptomatic p.p.p. at flowering	Average DS.Gf score	Average proportion of DI.Flw (%)
AMP	13.2 (11.8–16.5)	4.6 (1.6–7.3)	3.68 (2.9–3.9)	31.50
BMP	14.6 (12.9–16.2)	5.5 (2.9–6.8)	3.22 (3.02–3.66)	34.60

**Figure 1:**
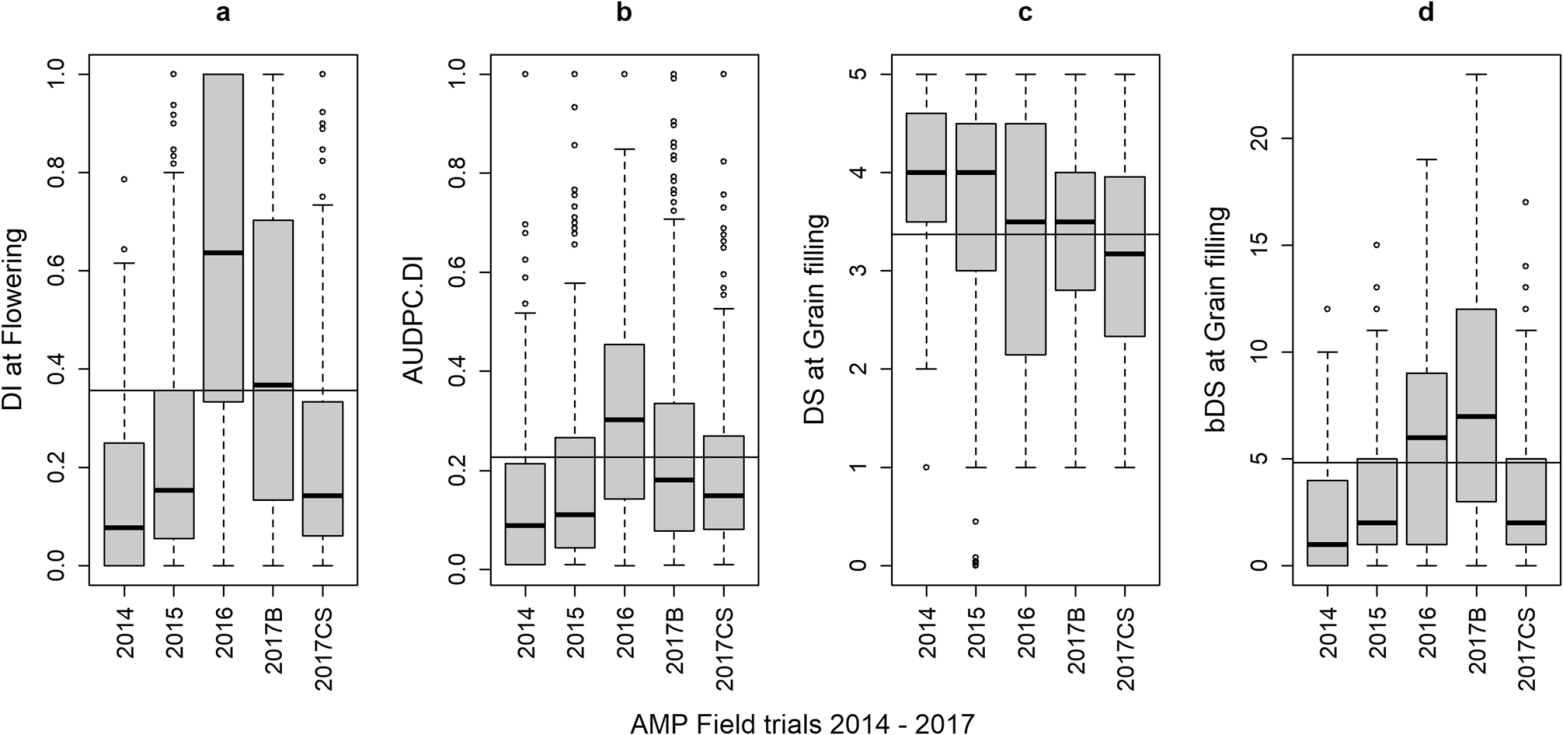
SVW Disease Descriptors of the AMP: Boxplots representing position measurements for the raw-data of four DDs scored across AMP’s five FTs. (**a**) Disease Incidence at Flowering (DI.Flw); (**b**) AUDPC.DI; (**c**) Disease Severity at Grain Filling onset (DS.Gf: mean score per plot); (**d**) Binomial Disease Severity at Grain filling onset (bDS.Gf). Seasons are named by their sowing year.

All FTs conducted with the AMP showed differences in disease levels, regardless of the phenotypic variable under analysis. The FTs conducted in Balcarce (South East of Buenos Aires Province) held values of DI.Flw and AUDPC.DI markedly above the overall mean in season 2016/17 and below it in 2014/15 and 2015/16. Altogether, AUDPC.DI and DI.Flw showed a similar pattern (Fig. 1). By contrast, disease descriptors for the FT conducted at Coronel Suarez (AMP—FT 2017CS, South West of Buenos Aires Province) were below the overall mean for DI.Flw (CS: 2.43; overall 4.16 p.p.p.), AUDPC.DI (CS: 0.199; overall: 0.227) and DS.Gf (CS: 3.06; overall: 3.68) (Fig. 1). In FTs where DI.Flw was below the overall mean, the average DS.Gf tended to be higher. This result was expected considering that in environments with lower disease pressure the infected ILs are the most susceptible ones.

**Figure 2:**
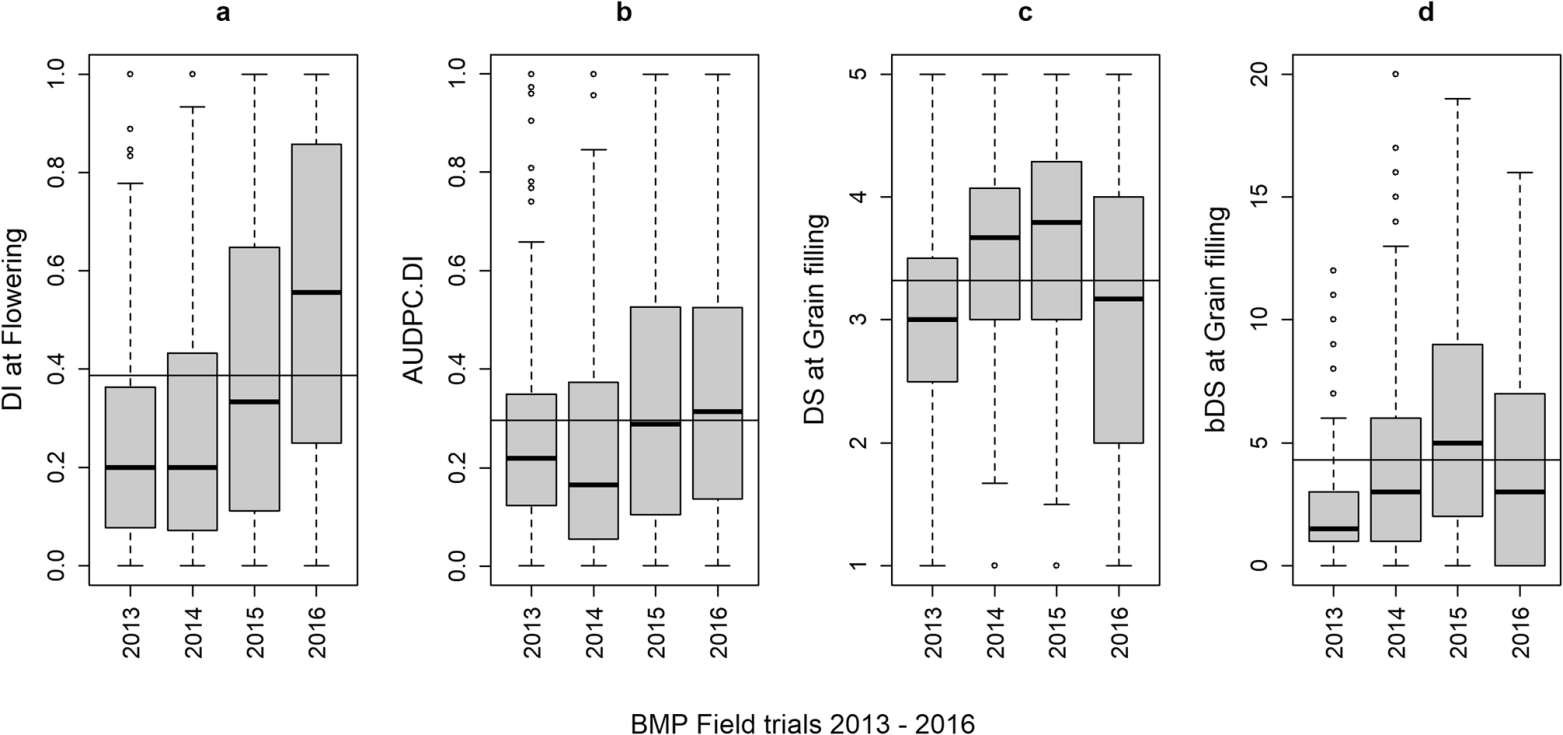
SVW Disease Descriptors of the BMP: Boxplots representing position measurements for the raw-data of four DDs scored across four BMP’s FTs. (**a**) Disease Incidence at Flowering (DI.Flw); (**b**) AUDPC.DI; (**c**) Disease Severity at Grain Filling onset (DS.Gf: mean score per plot); (**d**) Binomial Disease Severity at Grain filling onset (bDS.Gf). Seasons are named by their sowing year.

Differences among FTs were also observed for the BMP. DI.Flw and AUDPC.DI values tended to increase progressively from the first to the fourth FT. DS.Gf values were above the overall mean during seasons 2014/15 and 2015/16 and below it in the remaining seasons, although this pattern changed when considering DS as a binary variable (Fig. 2).

### Model selection and IL’s phenotypic means

All the tested models detected significant differences among ILs in both MPs. Almost all of the analyzed DDs presented significant Genotype effects (*p* < 0.001), except for DInt at the first evaluation date in the AMP (DInt.1). The selected model for further analysis was the Row-Column design with *Genotype* × *Season* interaction (*GS, *Eq. 3), because it showed the lowest Akaike Information Criteria values (AIC). The distribution of adjusted means across ILs for main DDs on each MP are presented in Table 2. It is worth noting that DS.Gf is evaluated only on infected plants and therefore minimum values are the average scoring of symptomatic plants in the plot. The phenotypic classes for every DD (DGC-classes), derived from post-hoc adjusted means comparison, are presented in Supplementary Tables S1 and S2 for each MP, respectively. The DGC procedure^34,35^ classified ILs into a maximum of three classes depending on the DD (Table 2).

**Table 2:** Distribution of adjusted phenotypic means for four disease descriptors on each MP.

AMP	DI.Flw	AUDPC.DI	DS.Gf normalized (raw)	bDS.Gf	BMP	DI.Flw	AUDPC.DI	DS.Gf normalized (raw)	bDS.Gf
Min	0.019	0.022	− 0.933 (1.7)	0.014	Min	0.027	0.011	− 1.632 (1.6)	0.006
Max	0.874	0.557	1.678 (4.9)	0.922	Max	0.948	0.586	1.719 (4.7)	0.927
Adj. Mean	0.317	0.169	0.421 (3.06)	0.355	Adj. Mean	0.327	0.245	− 0.074 (3.22)	0.264
SD	0.236	0.118	0.571	0.267	SD	0.206	0.140	0.658	0.205
DGC classes	3	2	2	3	DGC classes	3	2	2	3

Adjusted means for DS.Gf were estimated on the transformed variable, the average in the ordinal DS-scale is presented between brackets.

### Inbred lines with contrasting phenotypes

Taken together, the adjusted phenotypes for the four DDs indicate that a broad range of disease resistance responses are consistently represented in both MPs. In the AMP, ILs showed a broad spectrum of SVW symptomatic diversity, not completely measurable by the DDs, and a broad range of resistance levels. Considering the four selected DDs, 24 ILs were distributed among the top ten positions, whereas 23 were among the bottom ten positions. Within the most resistant ILs, PMA159, PMA41, PMA26, PMA89 and PMA24 occupied the top ten of three DDs, with a remarkable resistance for both DI and DS. Among these top 24 ILs, some ILs preferentially occupied the top ranks for DI (PMA51, PMA146, PMA152) or DS (PMA79, PMA46, PMA162, PMA64) derived descriptors (Supplementary Table S3).

In the AMP’s susceptible group, five ILs (PMA124, PMA31, PMA129, PMA122 and PMA44) ranked in the bottom ten of the four DDs. The highest level of DI.Flw and bDS.Gf occurred in IL PMA70 (probability of 0.87 and 0.92, respectively). As seen in the resistant group, some ILs exclusively ranked at the bottom of a particular DD. None of the parental lines of the BMP, both present in the AMP, were ranked on the extremes of any DD (Table 3).

**Table 3:**
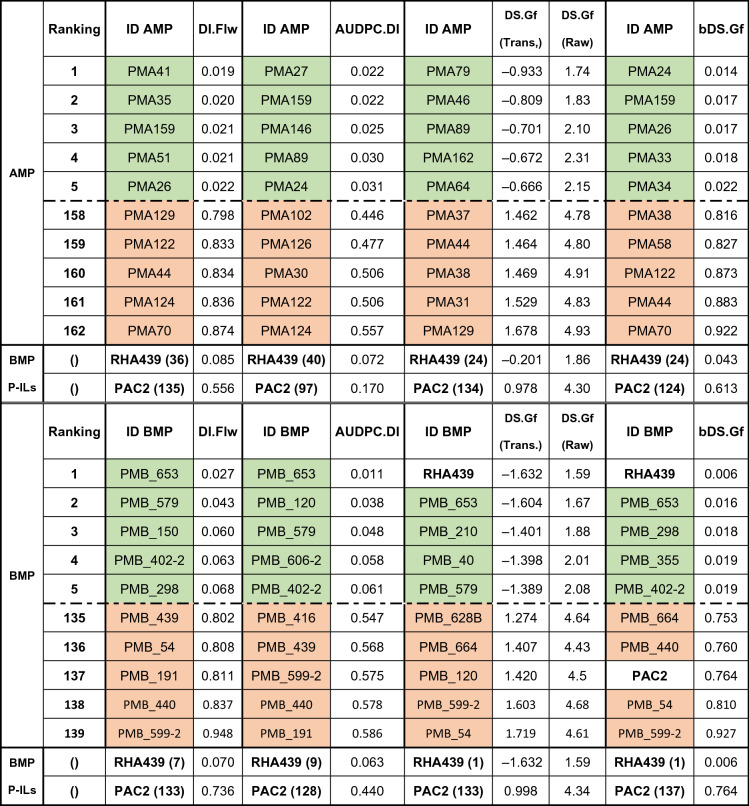
Top five and bottom five ILs and RILs for the four disease descriptors evaluated on each MP.

BMP's parental ILs (P-ILs) means and ranks. DS.Gf is presented transformed and as the average raw DS score of the IL.

In the BMP itself, a group of RILs consistently transgressed the SVW responses of the parental lines, both for resistance and susceptibility (Table 3 and Supplementary Table S4). In addition, 18 and 17 RILs distributed among DDs in the top and bottom ten positions, respectively. Within the resistant group of ILs, RHA439 was outperformed for DI but not for DS-related DDs. This IL was outperformed on DI related DDs by PMB_653, PMB_579, PMB_298 and PMB_402-2. Nonetheless, these RILs also ranked in top positions for DS related DDs.

In the susceptible group, RILs with worse performance than PAC2 occurred in the four DDs. Of the 17 RILs of the susceptible group, PMB_599-2, PMB_440, PMB_54, PMB_597 and PMB_191 were the most represented in the lower ranks of each DD. A relevant result to highlight is the positioning of RIL PMB_120 at the bottom-ten rank for DS.Gf, in contrast to its position in the top-ten rank for DI.Flw and AUDPC.DI.

### Phenotypic stability

**Figure 3:**
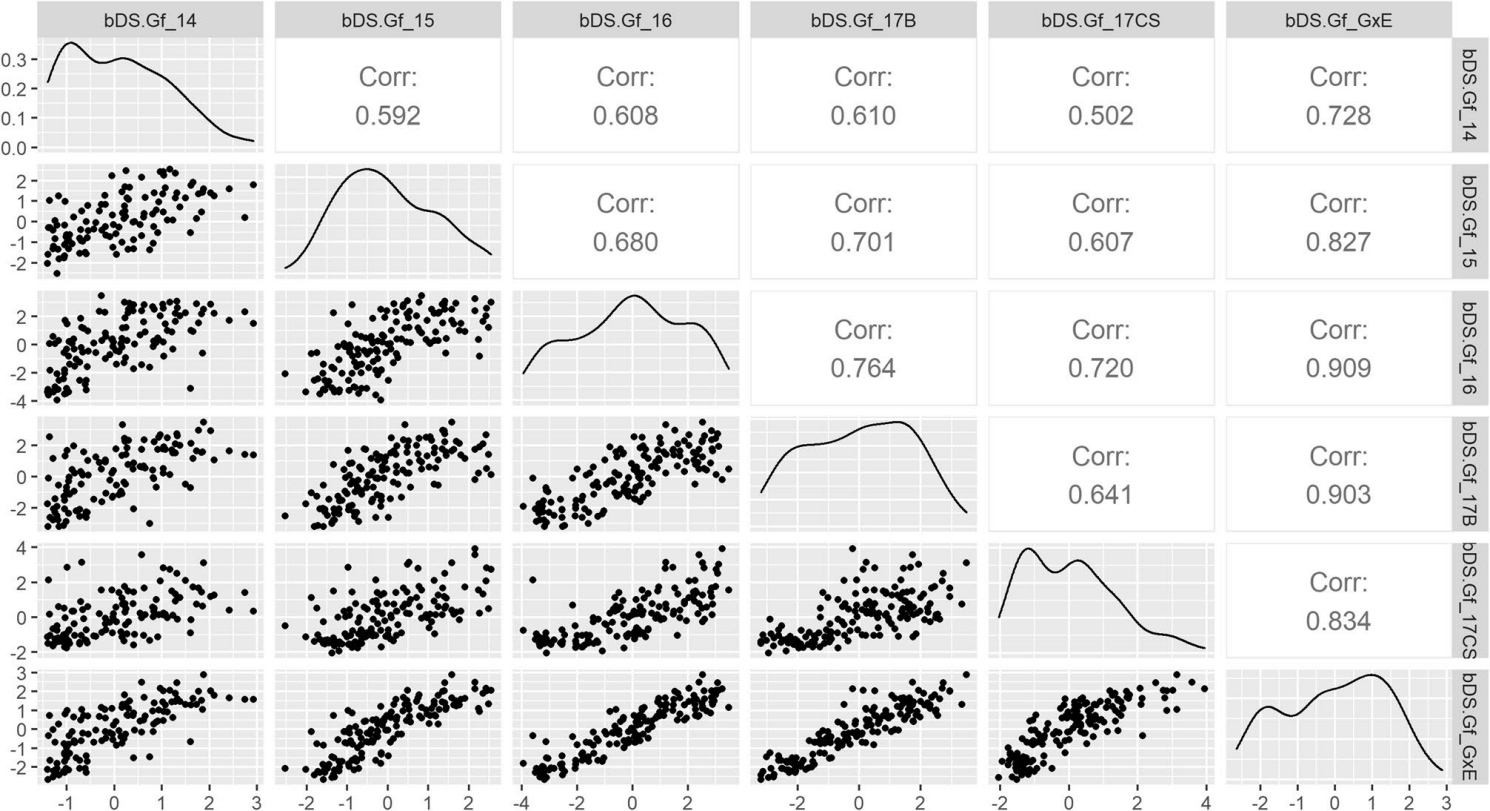
Pearson’s Correlations between FT BLUPS and G×E BLUPS for binomial-DS.Gf in the AMP. Below the diagonal: Scatter Plots of AMP-ILs’ BLUPs for bDS.Gf; Above the diagonal: Pearson’s correlation coefficients between AMP-ILs’ BLUPs for bDS.Gf (all *p* values < 0.01); Diagonal: Density plots showing the distribution of BLUPs frequencies. Seasons are named by their sowing year.

**Figure 4:**
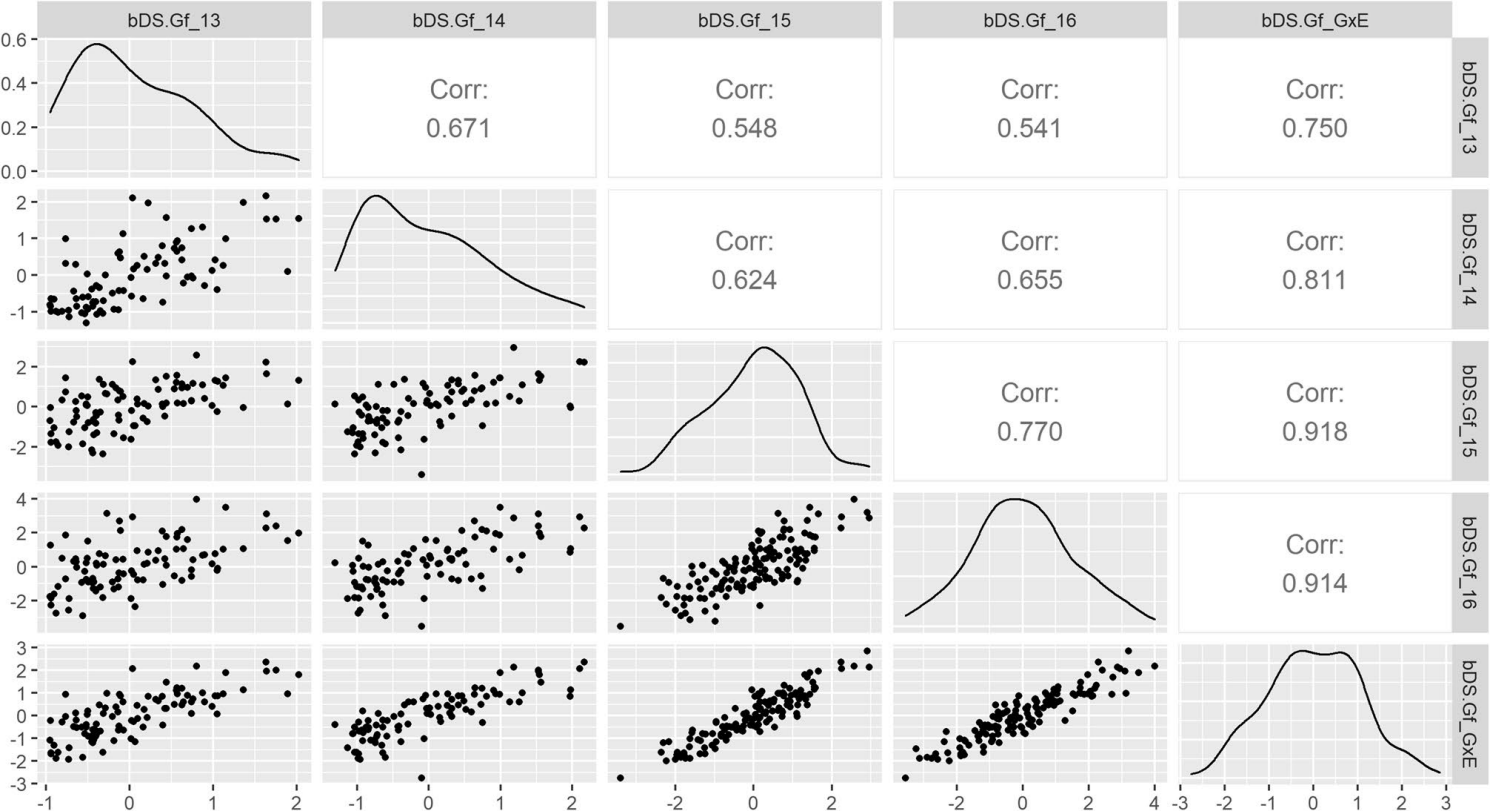
Pearson’s Correlations between FT BLUPS and G×E BLUPS for binomial-DS.Gf in the BMP. Below the diagonal: Scatter Plots of BMP-RILs’ BLUPs for bDS.Gf; Above the diagonal: Pearson’s correlation coefficients between BMP-RILs’ BLUPs for bDS.Gf (all *p* values < 0.01); Diagonal: Density plots showing the distribution of BLUPs frequencies. Seasons are named by their sowing year.

To analyze the phenotypic stability of ILs across different FTs and to have an insight into the variation attributable to Genotype by Environment interactions (G×E), we calculated Pearson’s correlation coefficients between Best Linear Unbiased Predictors (BLUPs) of individual FTs, and between these and the G × E adjusted BLUPs, for the main four DDs (Supplementary Figs. S1 and S2). The descriptor bDS.Gf was selected as example, because it represents the overall pattern of variation. This variable gives a good estimation of disease impact by jointly considering the amount of plants affected by SVW at the last evaluation date and symptom intensity, both for the AMP and the BMP (Figs. 3 and 4, respectively). For the AMP, the average correlation coefficient between individual FT BLUPs for bDS.Gf was of 0.643. The highest correlation for bDS.Gf was between the last two FTs of EEA-INTA Balcarce FT-2016 and FT-2017 (0.764), whereas the lowest was between the FT-2014 and the FT-2017CS (0.502) (Fig. 3). FT-2014 also presented a skewed distribution towards lower bDS.Gf levels (refer to frequency density-curve, Fig. 3). The correlation between the FTs conducted at Coronel Suarez and at Balcarce on 2017 was similar than the average (0.641), thus indicating a stable response of the AMP against different inoculum sources within the same season.

Interestingly, the G×E adjusted BLUPs, estimated by models including G×E interactions, showed the highest correlations with the individual FT BLUPs. This result indicates the usefulness of these models to deal with the proportion of phenotypic variation derived by G×E interactions. The lowest correlation coefficients occurred with FT 2014, which underlines the lower representability of this FT for disease resistance estimation. In agreement with its skewed frequency distribution towards lower bDS.Gf values, the FT-2014 showed the lowest correlation with the G×E adjusted BLUPs.

In the BMP, the average correlation coefficient between trials for bDS.Gf was of 0.64. The highest correlation coefficient for bDS.Gf was between FT-2015 and FT-2016 (0.77), whereas the lowest was between the first and the last FTs (0.541) (Fig. 4). As FT-2014 in the AMP, the BMP’s first FT showed a skewed frequency distribution towards lower levels of bDS.Gf (Fig. 4). As it was the case for the AMP, FTs’ correlations with the G×E adjusted BLUPs were the highest.

### Principal components analysis

To assess the informativeness for Principal Components Analysis (PCA) of the 18 DDs scored in this study, we computed Pearson’s correlations among the IL’s G×E adjusted means on each MP. All coefficients were significant, positive and rather high. Values were always above 0.5 or 0.6 for the AMP and BMP, respectively. Coefficients above 0.95 occurred between some contiguous evaluation dates for DI, DInt and bDS (Supplementary Figs. S3 and S4). Altogether, the collinearity observed confirms the utility of PCA for summarizing the information held in both MPs at a multivariate level.

**Figure 5:**
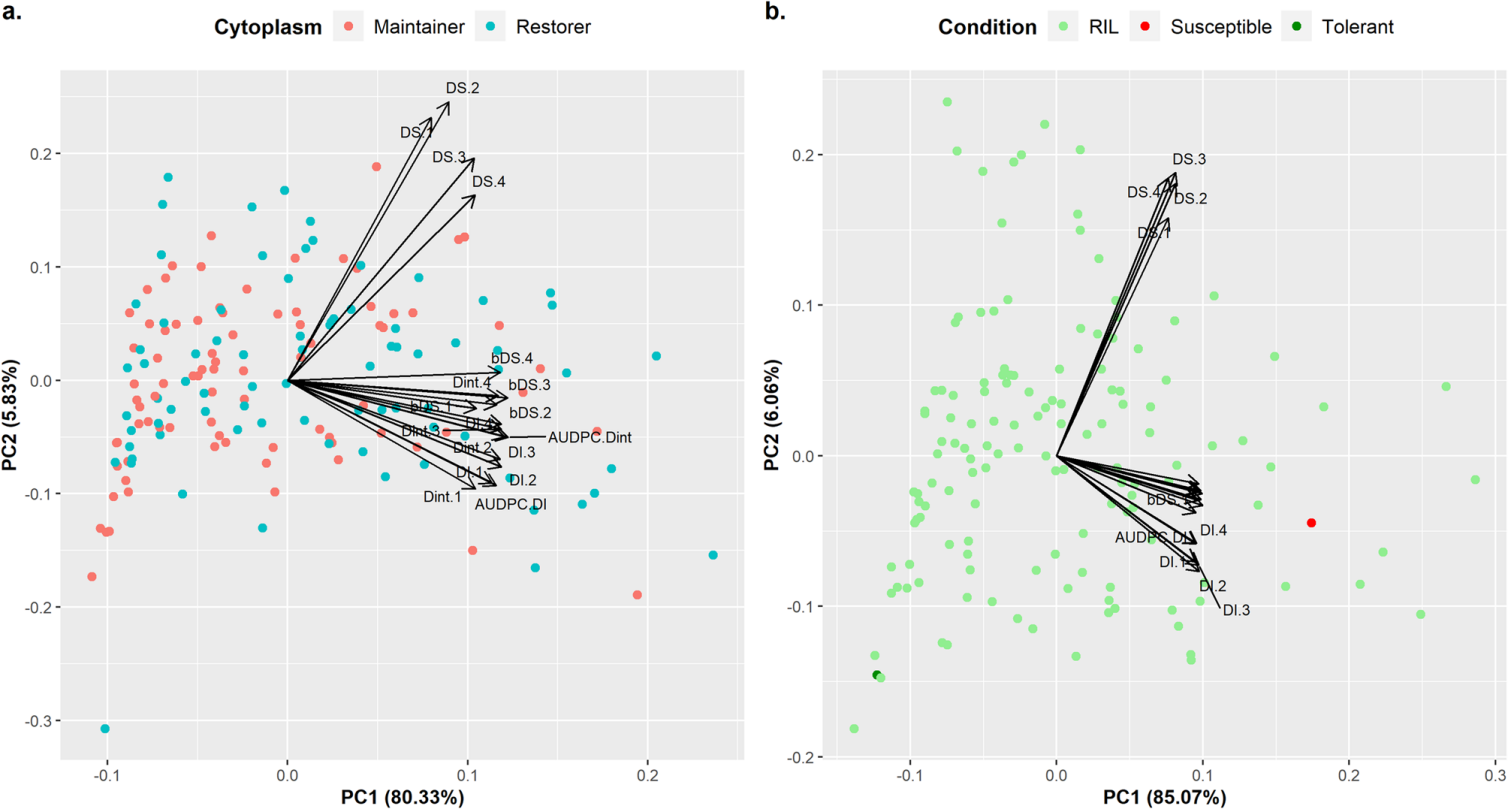
(**a**) Biplot of PCA of the AMP. Colors represent the cytoplasmic male sterility status of the 162 ILs; (**b**) Biplot of PCA of the BMP. Colors indicate the parental ILs and the RILs.

Considering the AMP’s PCA, the first two Principal Components (PC1 and PC2) explained 86.1% of the total variance (PC1: 80.3%; PC2: 5.83%; Supplementary Table S5). PC1 is mainly defined by DI-related variables, whereas PC2 is linked to DS. Lower values of PC1 are associated with higher levels of SVW-BSR, whereas lower values of PC2 are associated with lower DS scores. Considering AMP’s PC1, the top five tolerant lines were PMA89, PMA24, PMA22, PMA91 and PMA46 and the most susceptible five were PMA124, PMA129, PMA70, PMA122, PMA44. No associations were detected between Cytoplasmic Male Sterility Status and SVW-BSR (Fig. 5a). Biplots presenting ILs partitioned by Cycle Length and by Branching are depicted in Supplementary Figures S5 and S6, respectively.

In the BMP, the first two PCs explained 91.1% of the total multivariate variance (PC1: 85.1%; PC2: 6,1%; Supplementary Table S6). Concordantly with AMP, PC1 is mainly defined by the DI-driven DDs, whereas PC2 is related to DS-driven descriptors (Fig. 5b). The top five tolerant lines considering BMP’s PC1 were PMB_653, PMB_579, RHA439, PMB_255 and PMB_298, whereas the most susceptible five were PMB_599-2, PMB_54, PMB_440, PMB_191 and PMB_439.

### Clustering

Euclidean distances were calculated between ILs using the 18 DDs adjusted means in both MPs. The most likely number of clusters was of four (K = 4) in both cases. Further hierarchical and non-hierarchical clustering analyses based on distances and cluster number allowed the distinction of a fourth phenotypic group that was not apparent when analyzing the data on a single variable basis (DGC procedure detected between 2 and 3 classes). The K-mean method, presented here, generated more evenly represented groups for both MPs (Tables 4, 5 and Supplementary Figs. S7 to S10).

**Table 4:** AMP Cluster’s means and standard deviations, between brackets, for each DD.

AMP K-means	DI.Flw	AUDPC.DI	DS.Gf	bDS.Gf	ILs proportion
1	0.08 (0.05)	0.06 (0.03)	− 0.11 (0.37)	0.08 (0.06)	58 (36%)
2	0.3 (0.1)	0.15 (0.05)	0.44 (0.34)	0.34 (0.12)	51 (31%)
3	0.52 (0.1)	0.26 (0.06)	0.89 (0.28)	0.63 (0.1)	34 (21%)
4	0.73 (0.09)	0.39 (0.08)	1.15 (0.31)	0.75 (0.11)	19 (12%)

Proportion of ILs per group defined by K-means ordered by resistance level (1: Highly tolerant, 4: Highly susceptible).

**Table 5:** BMP Cluster’s means and standard deviations, between brackets, for each DD.

BMP K-means	DI.Flw	AUDPC.DI	DS.Gf	bDS.Gf	RILs proportion
1	0.13 (0.06)	0.11 (0.05)	−0.7 (0.49)	0.07 (0.04)	43 (31.2%)
2	0.25 (0.09)	0.2 (0.07)	− 0.01 (0.47)	0.19 (0.08)	41 (29.7%)
3	0.48 (0.08)	0.35 (0.07)	0.22 (0.39)	0.41 (0.1)	43 (31.2%)
4	0.76 (0.09)	0.52 (0.06)	0.95 (0.43)	0.71 (0.12)	11 (8%)

Proportion of RILs per group defined by K-means ordered by resistance level (1: Highly tolerant, 4: Highly susceptible).

### Heritability

The study of broad sense heritability (H^2^, Table 6) revealed that the average H^2^ coefficients for the four DDs were rather high in both MPs (H^2^ > 0.34 AMP; H^2^ > 0.24 BMP). In comparison to the remaining DDs, DI.Flw showed the lowest average H^2^. Furthermore, DS.Gf (H^2^ = 53.2) and AUDPC.DI (H^2^ = 43.87) had the highest heritability coefficients for the AMP and for the BMP, respectively. As expected, the AMP showed higher levels for each H^2^ estimate and larger standard deviations than the BMP. The BMP denoted a progressive increase in the H^2^ estimates for most of the variables along FTs. In the AMP, this response was evident only for DI.Flw, considering only FTs performed in Balcarce. In general, the H^2^ estimates obtained at Coronel Suarez were lower than those of Balcarce on the same year. For most DDs, the environments with higher H^2^ coefficients were FT-2016 and FT-2017B for the AMP, and FT-2015 and FT-2016 for the BMP.

**Table 6:** Broad sense heritability coefficients for SVW-DDs of both Mapping Panels across field-trials.

	DI.Flw	AUDPC.DI	DS.Gf	bDS.Gf
**AMP**				
2014	32.37*	71.39*	70.87*	36.00*
2015	22.29	34.23	35.69	30.93
2016	43.02	53.76	72.58	59.77
2017B	47.08	62.25	35.94	49.21
2017CS	26.65	41.77	50.91	39.76
Overall average	34.28	52.68	53.20	43.13
Overall SD	10.56	15.02	18.01	11.46
Overall range	22.29–47.08	34.23–71.39	35.69–72.58	30.93–59.77
**BMP**				
2013	16.84	8.27	4.18	17.60
2014	23.84	74.95	46.55	25.90
2015	28.33	49.13	50.19	31.11
2016	27.02	43.13	50.87	39.27
Overall average	24.01	43.87	37.95	28.47
Overall SD	5.14	27.46	22.59	9.10
Overall range	16.84–28.33	8.27–74.95	4.18–50.87	17.6–39.27

Overall average, standard deviation (SD) and range are presented below for each DD across field trials.

*Indicates field trials sown under a single-replicated augmented design.

## Discussion

SVW has become an important concern for sunflower breeding worldwide. To date, however, broad-spectrum resistance sources are not yet available, and little is known about the genetic determinants of defense responses.

Here we present the first large scale evaluation of germplasm by using two different MPs representative of biparental and association QTL-mapping approaches. Our research consisted of nine FTs under field-growing conditions within the region with the highest prevalence of SVW in Argentina. The FTs were performed in infested fields harboring a wide diversity of *Vd* isolates collected from the main sunflower producing region of the country. These trial conditions have no precedent in terms of the number of accessions under field evaluation, the number of FTs performed, the genetic diversity of the MPs and the natural inoculation method implemented. Thus, the conditions of the plant-pathogen interaction are comparable to those of sunflower production under a high inoculum pressure. In addition, our experimental design allowed us to estimate, for the first time, the heritability of disease resistance based on different DDs.

Considering the DDs used here for assessing SVW resistance, the six-level DS scale used^4,20^ slightly differs from others described previously by Bertero De Romano et al.^17,36^. As well, it differs even more from the DS scales implemented more recently, consisting of 9–10 levels^18,37,38^. In addition, our characterization spanned from R1 to R7 phenological stages^39^, whereas previous evaluations focused on the R5–R6 and were only exceptionally extended to R9 stages^4,17,36–38,40^. The extension of the evaluation resulted in a broader picture of SVW resistance profiles and allowed the assessment of the relationship between early symptoms and SVW final impact. This approach provides an interesting phenological window to scope for associated QTL in further studies.

Following the criterion of potential impact on yield and maximization of the DD informativeness, we selected four DDs to illustrate IL responses (DI.Flw, AUDPC.DI, DS.Gf and bDS.Gf). Since the critical stages for potential grain-yield definition are flowering and grain filling onset, three DDs focused on these stages assessing DI, DS and the combination of both relative to a tolerance threshold (DI.Flw, DS.Gf and bDS.Gf, respectively). AUDPC.DI, while integrating all DI scores through time, gives a cumulative resistance profile from early stages of each IL, giving higher values to ILs presenting symptomatic plants earlier. The bDS.Gf descriptor seemed to be most suitable for quick disease assessment since it provided a weighted estimation of DS.

The breakage of resistances and the appearance of new *Vd* pathogenic races have underscored the need for BSR sources in sunflower^12–14,27,41^. The inoculum load measured in the infested field of EEA INTA Balcarce (circa 900 CFU/g, Supplementary Table S7), which is known to harbor a wide diversity of *Vd* isolates, is four times higher than the reported by Erreguerena et al.^42^. In that report, 70–260 CFU/g rendered SVW-DI levels from 40 to 60%. The comparison with our results emphasizes the challenging conditions under which sunflower MPs were characterized in our study. Therefore, the resistant ILs identified here emerge as a promising resource for SVW-BSR.

Phytopathologically, Argentinian *Vd*-races affecting sunflower differ from the emerging North American race 2^12^. In a previous study of our group, a PCR molecular analysis of VArg1, VArg2 and an isolate from USA^43^ characterized these races as molecular race-2, due to the absence of the Ave1 effector^44,45^. The results of this previous study were developed together with race-specific resistance tests over a subset of ILs under controlled conditions^43^.

In this sense, Martín-Sanz et al.^14^ classified an Argentinian isolate within the group of European race V2-WE by molecular and virulence tests. Altogether, this suggests that the BSR sources identified here might be useful for conferring resistance to sunflower- affecting-races around the world.

Despite the higher genetic diversity held in the AMP, this population showed DI and DS averages similar to those of the BMP. Standard deviations, however, were slightly higher for the AMP. The AMP includes restorer and maintainer inbred lines exhibiting resistance responses to SVW. It’s overall genetic diversity is similar to that from other sunflower germplasm panels from public breeding programs of Canada, France and USA, while containing the singularities of the Argentinian germplasm^32^. The parental lines of the BMP, RHA439 and PAC2, are two public restorer ILs derived from the USDA and INRA sunflower breeding programs, respectively. Both have shown partial resistance to Sclerotinia Head Rot^46–48^, but have no reports on SVW response. The Plant Pathology Department of INTA-Balcarce screened the on-field SVW resistance of a set of ILs and defined RHA439 as highly tolerant and PAC2 as highly susceptible to Argentinian *Vd*-races. Each IL respectively grouped in resistant and susceptible clusters of the AMP’s clustering analyses, although none of these occupied the top ranks of any of the four DDs (Table 3 and Supplementary Table S3).

In the BMP, a group of RILs consistently outperformed the SVW responses of the parental lines (Table 3). This finding indicates that a substantial proportion of the inheritance of SVW resistance is explained by polygenic components. The observed transgressive segregation, both in resistance as in susceptibility, indicates that recombination of genetic factors controlling SVW response was achieved during BMP development. RHA439 was outperformed mainly in DI-DDs by RILs, but not in DS-DDs. This result suggests a more complex genetic architecture underpinning DI-related traits. This was also seen in *V. dahliae* wilts affecting other crops such as cotton^49–51^, strawberry^52^, *Medicago truncatula*^53^ and olive^54^.

Soil-borne fungal diseases are hard to evaluate. Particularly, the spatial overlapping of host and pathogen populations is a critical subject for determining patterns of disease occurrence and dynamics^38^. The level of disease virulence observed in the AMP varied across FTs. The low level of DI observed in the 2014 FT could be the result of sowing the trial on the margins of the infested field, where the inoculum density might have been lower. Alternatively, late sowing dates may have also affected SVW infection in this FT (Supplementary Table S8)^20,55,56^. By contrast, the BMP was sown on optimal dates at each FT and occupied central locations in the SVW-testing field, therefore ensuring optimal conditions for SVW infection and evaluation. Despite these difficulties, the levels of DI and DS observed in both MPs allowed the identification of significant differences in SVW-resistance among genotypes in all FTs. In this sense, the Row–Column experimental design contributed in accounting for spatial variability to weight the ILs’ SVW-resistance.

Integrating the information of the different FTs into a single adjusted mean may help to obtain robust measures useful to characterize ILs. For example, models including the G×E random effect showed the best fit to the data for both the AMP and BMP. As observed for the raw data, the AMP and the BMP showed similar adjusted means and standard deviations for the four selected DDs. Consistently with the fit of the G×E models, the multi-environment BLUPs yielded high correlations with those of the different FTs. Furthermore, the FTs with highest H^2^ coefficients presented the highest Pearson’s correlation values.

Interestingly, FT BLUP correlations observed for DI related DDs in the AMP between FT-2017B and FT-2017CS were higher than some observed within the same location analyzed between years. This suggests that climatic variables may have a larger effect on disease responses than expected. Moreover, these two testing fields are known to differ in their inoculum composition and virulence, according to INTA’s sunflower commercial-hybrids resistance comparison trials performed in these testing fields over the last 20 years (Carolina Troglia, pers. comm.). This notion supports the BSR displayed by the best performing ILs of the AMP. Despite the significant G×E effects, the high correlation coefficients observed among FTs indicate that the ranking of ILs was consistent across years for both MPs. Furthermore, the alternation of the groups of ILs occupying the top ranks for each DDs (Supplementary Tables S3 and S4) highlights the polygenic architecture of SVW resistance. This suggests the presence of specific genetic factors involved in SVW DI and DS, respectively.

Overall, the four main DDs displayed moderate to high heritability (Table 6). The estimated values for both populations encompassed a similar range of heritability values showing robust results, except for FTs 2013 (BMP) and FT 2014 (AMP). The heritability observed in FT 2014 (AMP) could have been biased by the unfavorable environmental and experimental conditions for the assessment of the disease on this particular trial, as mentioned above. DS.Gf showed higher overall-H^2^ coefficients than DI.Flw, despite the larger dispersion seen in DS.Gf. The descriptor bDS.Gf rendered higher H^2^ coefficients than DI.Flw, but showed similar dispersion levels. This resulted in a higher accuracy for phenotypic selection. Hence, bDS.Gf yielded a more efficient DD for selecting tolerant germplasm. As expected on the basis of their genetic diversity, the AMP’s H^2^ estimators were consistently higher than those of the BMP.

Considering individual FTs, DI.Flw showed sequentially increasing levels of H^2^ over the years in both MPs. This finding indicates that increments in inoculum density, whether because of location within the infected field or microsclerotia accumulation, reduced environmental variance and allowed a better estimation of genetic-variation effects on the phenotype. Indeed, the last FTs that were carried out at EEA-INTA-Balcarce displayed the highest heritability estimates for both MPs. Thus, these estimates may be considered as the most reliable for future genotype–phenotype association studies. These heritability values are promising for future mapping of QTLs defining SVW-resistance.

Although the description of ILs’ behavior against SVW was mainly focused on the four selected DDs, all 18 DDs were used to examine the relationship among ILs through ordination and clustering analyses. PCA successfully translated the high positive correlations observed between DDs to synthetic variables and therefore summarizes a high proportion of the observed phenotypic diversity in both MPs. PCA bi-plots reflected the skewness towards resistance seen at the univariate level in both panels (Fig. 5). In AMP’s bi-plots, partitioning ILs by their Cytoplasmic Male Sterility Status (Fig. 5a), Cycle Length (Supplementary Fig. S5) and Branching (Supplementary Fig. S6) allowed us to visualize putative associations between SVW resistance and these characteristics. Cytoplasmic status and branching showed no apparent relationship with SVW resistance level (Fig. 5a and Supplementary Fig. S6). Considering cycle length, a negative relationship is observed between DDs and long-cycle ILs. This is in agreement with the results reported by Fick and Zimmer^25^, who remarked the correspondence of lower DS-scores on ILs with longer cycle lengths (Supplementary Fig. S5).

In turn, the clustering analysis allowed us to define four phenotypic groups differing in their response to the pathogen on each MP. The identification of these clusters containing ILs with stable SVW-resistance constitutes a valuable resource for BSR breeding. Recently, we assessed a selection of ILs from the highly tolerant cluster in race-specific resistance trials and identified differential ILs for Argentinian VArg1 and VArg2 races^43,57^. In conclusion, the MPs studied here proved to harbor a large amount of genetic variability for the trait under study. In spite of the differences in genetic variability between a germplasm collection like the AMP and a biparental mapping panel as the BMP, the oligogenic nature of the trait seems to have allowed us to explore similar extents of phenotypic variation in both MPs. Moreover, although the analyzed DDs showed high correlations, we were able to detect different components of SVW resistance. In addition, ED extension over time allowed the identification of key evaluation points for maximizing SVW response variation. The SVW evaluation along different phenological stages allowed us to identify genetic resources with significant phenotypic differences throughout time. The identification of ILs with stable SVW-resistance and with outstanding behavior for specific DDs are valuable resources to be used for breeding purposes.

Finally, the relatively high heritability of the trait makes this genetic resource a suitable platform for future QTL mapping approaches in the search of genomic regions implied in SVW resistance.

## Methods

### Plant materials

Two mapping populations were evaluated for SVW resistance. A BMP composed of 139 RILs derived from the crossing of two public restorer lines, PAC2 and RHA439, susceptible and highly tolerant to *Vd* Argentinian races, respectively. The second is the INTA’s Association Mapping Population (AMP) described in Filippi et al.^30–32^, with the addition of 29 new accessions, thus reaching 164 ILs (83 restorers, 81 maintainers; Supplementary Table S9). All the ILs included in this study are preserved in the Active Germplasm Bank of EEA-INTA Manfredi. All plant studies were carried out in accordance with relevant institutional, national and international guidelines and legislation.

### Field-trials and experimental design

ILs from both MPs were grown under field conditions in artificially infested fields containing a diverse set of *Vd* strains, isolated from sunflowers grown along the main sunflower-growing region of Argentina. The BMP was evaluated in four FTs conducted at the EEA-INTA Balcarce, Balcarce, Buenos Aires (37°50′0″ S, 58°15′33″ W), from the growing seasons 2013/14 (F6) to 2016/17 (F10). The AMP was evaluated in five FTs, four at EEA-INTA Balcarce (2014/15 to 2017/18) and a fifth (2017/2018) at “El Cencerro” seed company, Coronel Suárez, Buenos Aires (37°25′52.0″ S, 61°51′32.5″ W).

The inoculum titter at EEA-INTA Balcarce’s evaluation field has been incremented by monocropping sunflower susceptible cultivars since 1997 and by soil tillage with crop debris. The *Vd* inoculum titter of EEA-INTA Balcarce’s infested field was estimated around 900 CFU per gram of soil in season 2015/16 (Supplementary Table S7), according to the procedure used by Erreguerena et al.^42^. El Cencerro’s infested field is regularly used for testing sunflower commercial hybrids for SVW-resistance, along with susceptible testers.

Plots of each FT consisted of a single 5 m row (± 0.5 m) disposed at an inter-row spacing of 0.7 m with 20 ± 5 plants. Each plot corresponds to an experimental unit. An Alpha-Lattice design in two replications was implemented for all FTs^58,59^. This model is transformable to a Row-Column design to enhance modelling precision under high spatial variability conditions. Susceptible internal controls were sown in plots across replications to estimate disease-spatial-variation. Supplementary Table S8 gives the number of ILs evaluated per FT along with other specifications, whereas Supplementary Figure S11 and Supplementary Table S10 depict the regular design of a SVW-phenotyping FT. FTs were conducted without any nutritional limitation, following the fertilization criteria used in the production fields of the region for fulfilling crop requirements. Watering was supplied when needed. Seeds were coated before sowing with commercial fungicides (APRON GOLD, 35 g Metalaxil-M) to prevent downy mildew (*Plasmopara halstedii*) infections. Mechanical and manual controls were implemented for weed management.

### Phenotyping for SVW-resistance

The DDs recorded to evaluate ILs were DI, DS, bDS, DInt, AUDPC.DI and AUPDC.DInt. DI and DS were scored at individual plant level. DI was assessed as the overall count of symptomatic plants per plot, whereas DS was scored using an ordinal scale of six levels, as follows: “0”, non-symptomatic plant, “1” plant with symptoms at basal leaves (under 20% of total leaf area, t.l.a.), “2” symptoms below middle leaves (20–40% t.l.a.), “3” symptoms reaching middle leaves (40–60% t.l.a.), “4” symptoms in upper leaves (60–80% t.l.a.) and “5”: a totally wilted plant (Supplementary Fig. S12).

DInt, bDS and AUDPCs were also calculated according to DI and DS scores. DInt is the per-plot weighted mean resulting from the multiplication of the frequencies obtained for each severity level and the score-level. The descriptor bDS accounts for infected plants with DS above “2” and any plant below this threshold is considered tolerant. AUDPC is the integration of the scores of a particular variable across the evaluation dates, according to the formula described by Shaner and Finney^60^ (Eq. 1).

1 $$AUDPC = \Sigma \left( {\left( {X_{i + 1} + X_{i} } \right)/2} \right)\left( {T_{i + 1} - T_{i} } \right)$$

where *X*_*i*_ is the proportion of symptomatic plants at the *i*th observation, whereas (*T*_*i*+1_ – *T*_*i*_) corresponds to the time elapsed between two observations (days).

Disease evaluation was performed weekly from early reproductive stages to grain filling onset, covering four evaluations per FT. Each ED was centered on the most frequent phenological stage among ILs at the time^39^. Therefore, each ED represents a particular phenological stage as follows: ED-1 = Floral initiation to Early floral bud growth (R1–R2); ED-2 = Floral growth to Pre-Flowering (R3–R4); ED-3 = Flowering (Flw, R5.1–R6); ED-4 = Grain filling (Gf, R7–R8)^39^. DDs are named both with number or acronym indistinctly (i.e.: DI.3 = DI.Flw). These combinations yielded 18 variables: 16 DD × ED combinations and two AUDPCs (for DI and DInt).

### Statistical analysis

For the estimation of IL’s mean phenotype for each SVW-DD, the statistical analysis at each evaluation date was boarded by Generalized Linear Mixed Models (GLMM)^61^. The selection of the right linking function when modelling was determined according to the statistical properties of each DDs. DI and bDS are discrete binomial variables and were modeled by logistic linear regressions. DS was transformed to a continuous variable by the Normal Scores method^62^, and modelled as a normally distributed variable by Linear Mixed Models (LMM). DInt and AUDPCs are continuous variables that were converted to relative proportions of the maximum score reached per plot and per FT, respectively, and modeled as gamma distributed variables. Several models were employed for obtaining IL’s adjusted phenotypic means and BLUPS^63^ for each DD, in both single and combined FTs (multienvironmental). These approaches allowed the comparison between them and the variance components analysis of random-effect models for DDs’ H^2^.

All of these models were fitted after the best model for each DD was found by comparing four models considering two experimental designs, Alpha-Lattice or Row–Column, and the inclusion of a random effect factor term considering G × E interaction under each design. For the Row–Column design, models were compared without (2) and with (3) the “*GS*” (Genotype-Season) interaction factor. The same models were tested for Alpha-Lattice design, which was obtained by excluding the factor “Column” (C) on (2) and (3).

Models 1 and 2 are defined as follows:

2 $$y_{ijkmn } = \mu + G_{i} + S_{j} + R\left( S \right)_{k\left( j \right)} + B\left( R \right)_{m\left( k \right)} + C\left( R \right)_{n\left( k \right)} + \varepsilon_{ijkmn}$$

3 $$y_{ijkmn } = \mu + G_{i} + S_{j} + R\left( S \right)_{k\left( j \right)} + B\left( R \right)_{m\left( k \right)} + C\left( R \right)_{n\left( k \right)} + GS_{ij} + \varepsilon_{ijkmn}$$

where $y_{ijkmn }$ is the adjusted DD score of the Inbred Line *i*, in Season *j*, under Replicate *k*, within Block *m* and Column *n*; *μ* is the overall mean of observations; *G*_*i*_ is the Inbred Line fixed effect (as random for BLUPs and H^2^ determination); *S*_*j*_ is the random effect of Season *j*; $R\left( S \right)_{k\left( j \right)}$ is the random effect of Replicate *k* within Season *j*; $B\left( R \right)_{m\left( k \right)}$ is the random effect of Block *m* within Replicate *k*; *C*_*n*_ is the random effect of Column *n* within Replicate *k*; and $\varepsilon_{ijkm}$ is the random residual term associated with observation $y_{ijkmn}$. In model (3), $GS_{ij}$ represents the random effect interaction between the Genotype fixed-effect factor and the Season random-effect.

The adjusted means were then subjected to multiple comparison tests using the DGC procedure^34,35^. BLUPs were calculated for each IL on every DD both, on each FT and across FTs, to evaluate the phenotypic stability of ILs across FTs and locations. Pearson’s correlation coefficients were used to compare BLUPs among FTs and models. Broad-sense heritability estimates were assessed for each DD considering the individual FT data. For binomial variables, residual variance components were estimated as described in Snijders and Bosker^64^. LMM and GLMM were built with the *lme4* package^65^ for the R statistical software^66^. Pearson’s correlations were estimated and visualized using the *GGpairs* R-package^67^.

Of the combination of DDs and EDs modeled, mainly four were considered to characterize ILs regarding SVW-BSR in a single-variant fashion. The criterion for selecting them accounted for agronomical issues and took into consideration SVW impact on yield components and the maximization of each DD descriptive capability. These four DDs were: DI.Flw (DI.3), AUDPC.DI, DS.Gf (DS.4) and bDS.Gf (bDS.4).

### Principal component analyses

PCA were carried out using the standardized adjusted means of DDs for each mapping population and the *prcomp* function of the *stats* package for the R statistical software^66^. Both MPs were analyzed with 18 DDs (DI, DS, DInt and bDS by four evaluation dates each and AUDPCs for DI and DInt). Results were visualized using the *ggfortify* R-package^68^.

### Clustering analyses

The clustering analysis was performed by estimating distance matrices for each MP from the adjusted means of DDs across FTs using the function *get_dist* of package *vegan* for R software^69^. The most likely number of clusters was defined using the function *fviz_nbclust* from the *factoextra* R-package^70^. Non-hierarchical clusters were estimated for each MP using the function “*kmeans*” of the R-package *stats*^66^. Finally, a hierarchical approach was performed by using the “*hclus*” function from the same package, by implementing different linkage methods and comparing them by their co-phenetic correlation coefficients with original distance matrices.

## Supplementary Information

Supplementary Information 1.

Supplementary Information 2.

## Data Availability

All data generated or analyzed during this study is included in this published article or in its Supplementary Information files.
