# Supplementary material for: On-field phenotypic evaluation of sunflower populations for broad-spectrum resistance to Verticillium leaf mottle and wilt
Source: Sci Rep. 2021 Jun 2;11:11644. doi: 10.1038/s41598-021-91034-4 (PMC8172943; doi:10.1038/s41598-021-91034-4)
Supplement: Supplementary file 1 — Supplementary Information 1. [file 41598_2021_91034_MOESM1_ESM.pdf]

On-field phenotypic evaluation of sunflower populations for broad-spectrum resistance to Verticillium leaf mottle and wilt

Juan F. Montecchia [1*], Mónica I. Fass [1], Ignacio Cerrudo [2], Facundo J. Quiroz [2], Salvador Nicosia [1], Carla A. Maringolo [2], Julio Di Rienzo [3], Carolina Troglia [2], H. Esteban Hopp [1,4], Alberto Escande [2], Julio González [5], Daniel Álvarez [6], Ruth A. Heinz [1], Verónica V. Lia [1,4*] and Norma B. Paniego [1]

[1] Instituto de Agrobiotecnología y Biología Molecular (IABIMO), Instituto Nacional de Tecnología Agropecuaria (INTA), Consejo Nacional de Investigaciones Científicas y Técnicas (CONICET), Hurlingham B1686IGC, Buenos Aires, Argentina.
[2] Instituto Nacional de Tecnología Agropecuaria (INTA). Estación Experimental Agropecuaria Balcarce, Buenos Aires, Argentina.
[3] Facultad de Ciencias Agropecuarias, Universidad Nacional de Córdoba, Córdoba, Argentina.
[4] Facultad de Ciencias Exactas y Naturales Universidad de Buenos Aires, Argentina.
[5] Instituto Nacional de Tecnología Agropecuaria (INTA). Estación Experimental Agropecuaria Pergamino, Buenos Aires, Argentina.
[6] Instituto Nacional de Tecnología Agropecuaria (INTA). Estación Experimental Agropecuaria Manfredi, Manfredi, Córdoba, Argentina.

*Correspondence and requests should be addressed to : montecchia.juan@inta.gob.ar (J.F.M.) and lia.veronica@inta.gob.ar (V.V.L.).

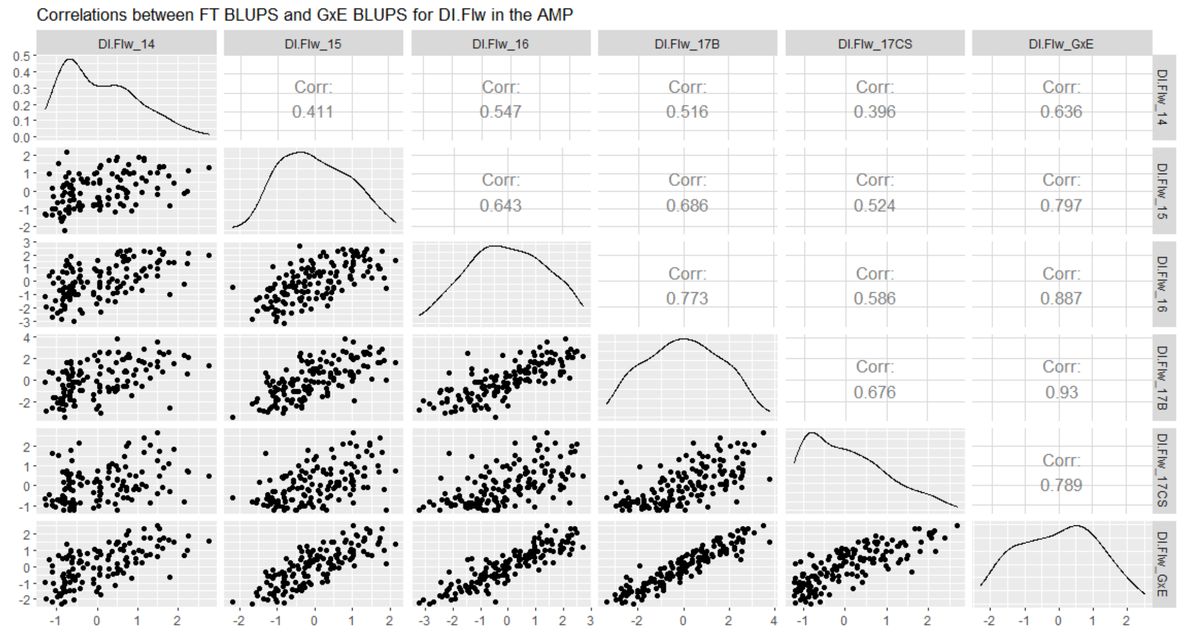

Supplementary Figure S1-a. Pearson's Correlations between FT BLUPS and GxE BLUPS for DI.Flw in the AMP. Below the diagonal: Scatter Plots of AMP-ILs' BLUPs for DI.Flw; Above the diagonal: Pearson's correlation coefficients between AMP-ILs' BLUPs for DI.Flw (all p-values < 0.01); Diagonal: Density plots showing the distribution of BLUPs frequencies. Seasons are named by their sowing year.

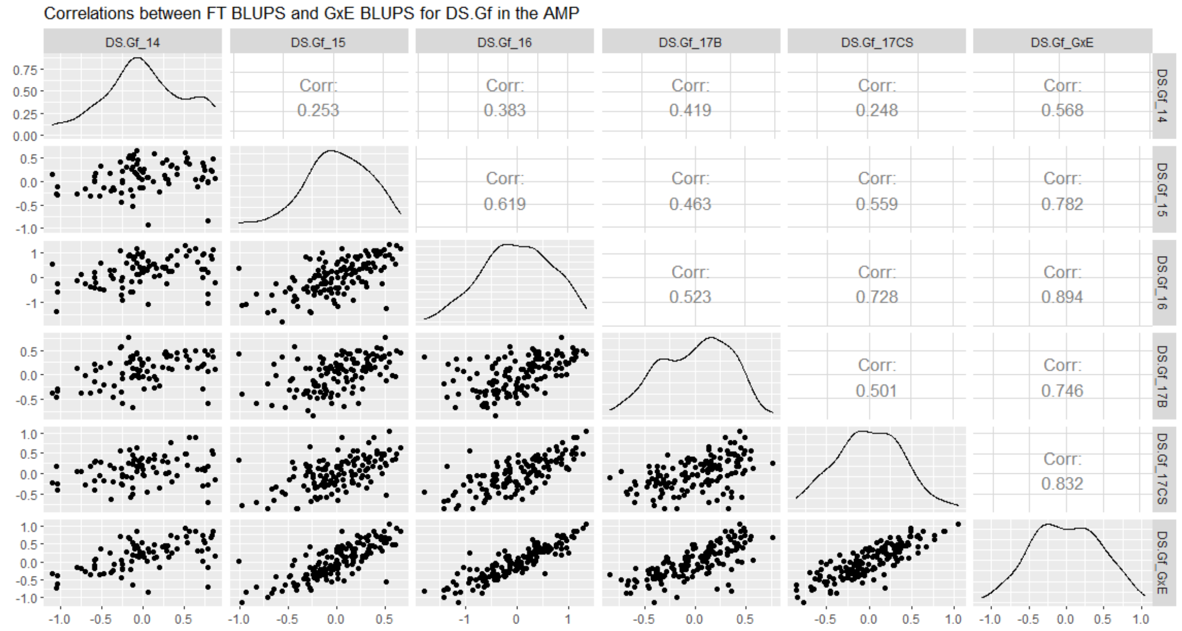

Supplementary Figure S1-b. Pearson's Correlations between FT BLUPS and GxE BLUPS for DS.Gf in the AMP. Below the diagonal: Scatter Plots of AMP-ILs' BLUPs for DS.Gf; Above the diagonal: Pearson's correlation coefficients between AMP-ILs' BLUPs for DS.Gf (all p-values < 0.01); Diagonal: Density plots showing the distribution of BLUPs frequencies. Seasons are named by their sowing year.

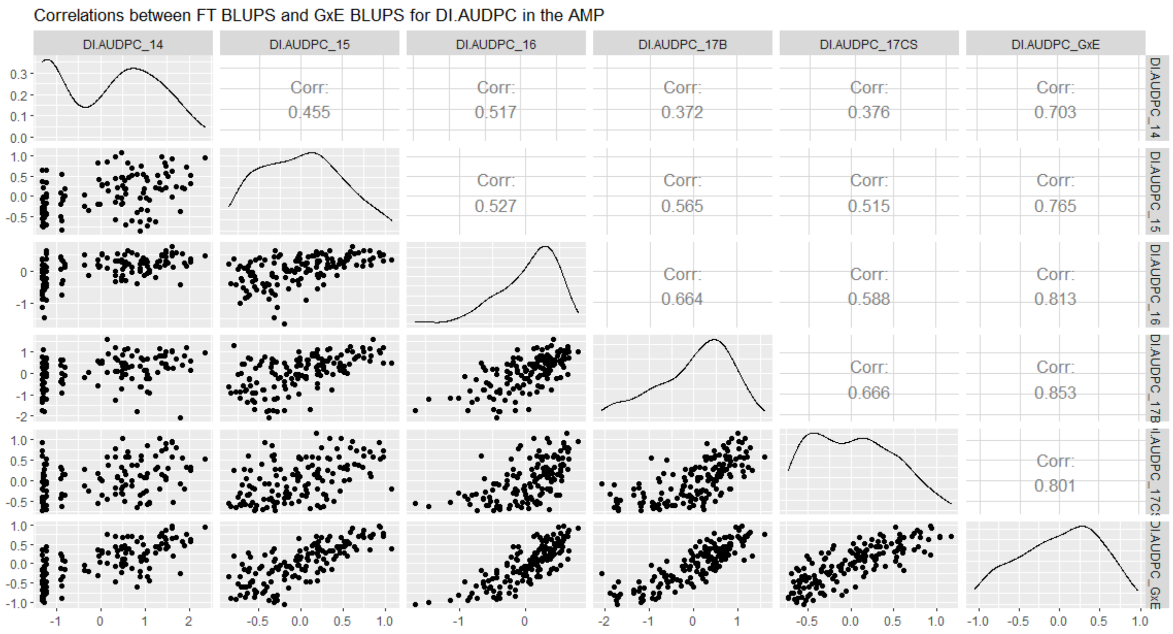

Supplementary Figure S1-c. Pearson's Correlations between FT BLUPS and GxE BLUPS for DI.AUDPC in the AMP. Below the diagonal: Scatter Plots of AMP-ILs' BLUPs for DI.AUDPC; Above the diagonal: Pearson's correlation coefficients between AMP-ILs' BLUPs for DI.AUDPC (all p-values < 0.01); Diagonal: Density plots showing the distribution of BLUPs frequencies. Seasons are named by their sowing year.

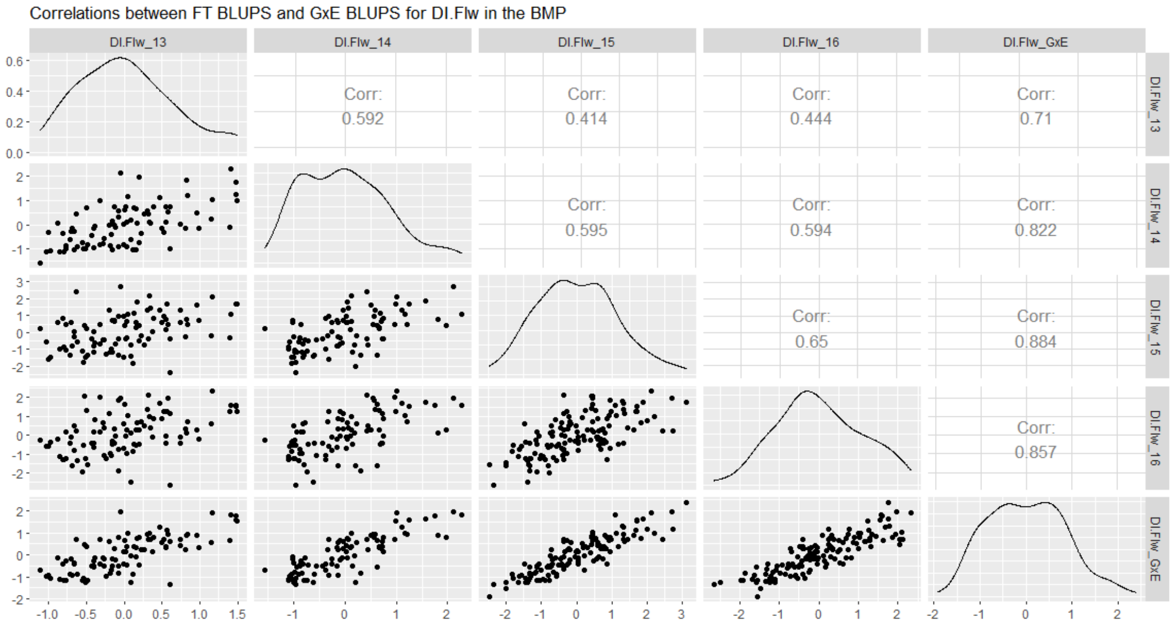

Figure S2-a. Pearson's Correlations between FT BLUPS and GxE BLUPS for DI.Flw in the BMP. Below the diagonal: Scatter Plots of BMP-RILs' BLUPs for DI.Flw; Above the diagonal: Pearson's correlation coefficients between BMP-RILs' BLUPs for DI.Flw (all p-values < 0.01); Diagonal: Density plots showing the distribution of BLUPs frequencies. Seasons are named by their sowing year.

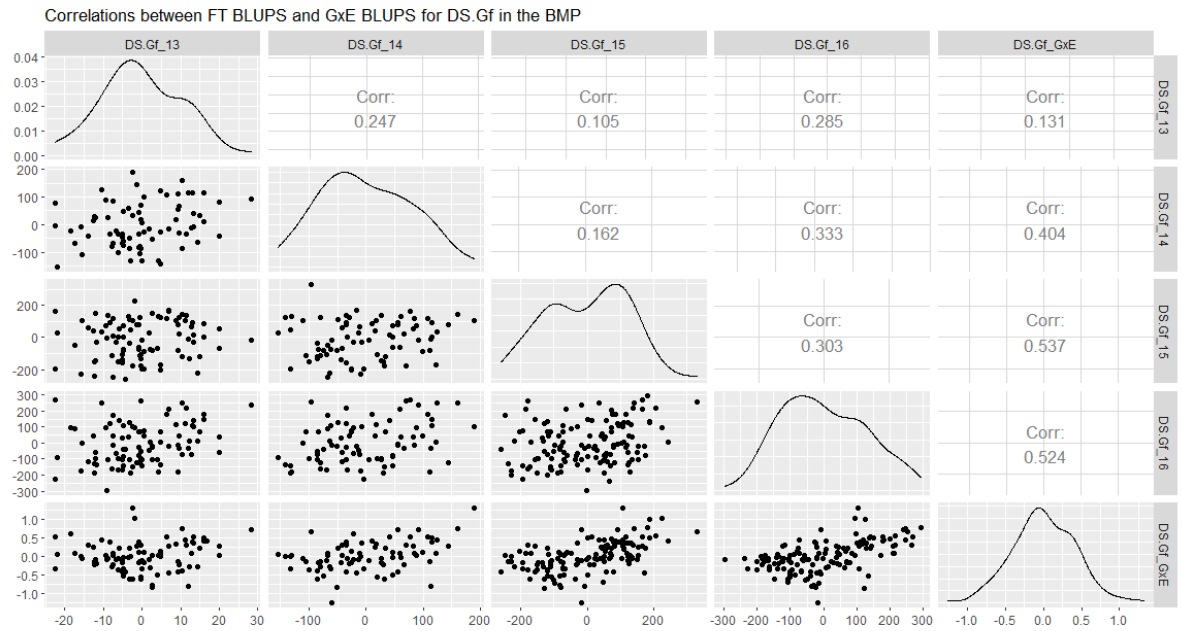

Figure S2-b. Pearson's Correlations between FT BLUPS and GxE BLUPS for DS.Gf in the BMP. Below the diagonal: Scatter Plots of BMP-RILs' BLUPs for DS.Gf; Above the diagonal: Pearson's correlation coefficients between BMP-RILs' BLUPs for DS.Gf (all p-values < 0.01); Diagonal: Density plots showing the distribution of BLUPs frequencies. Seasons are named by their sowing year.

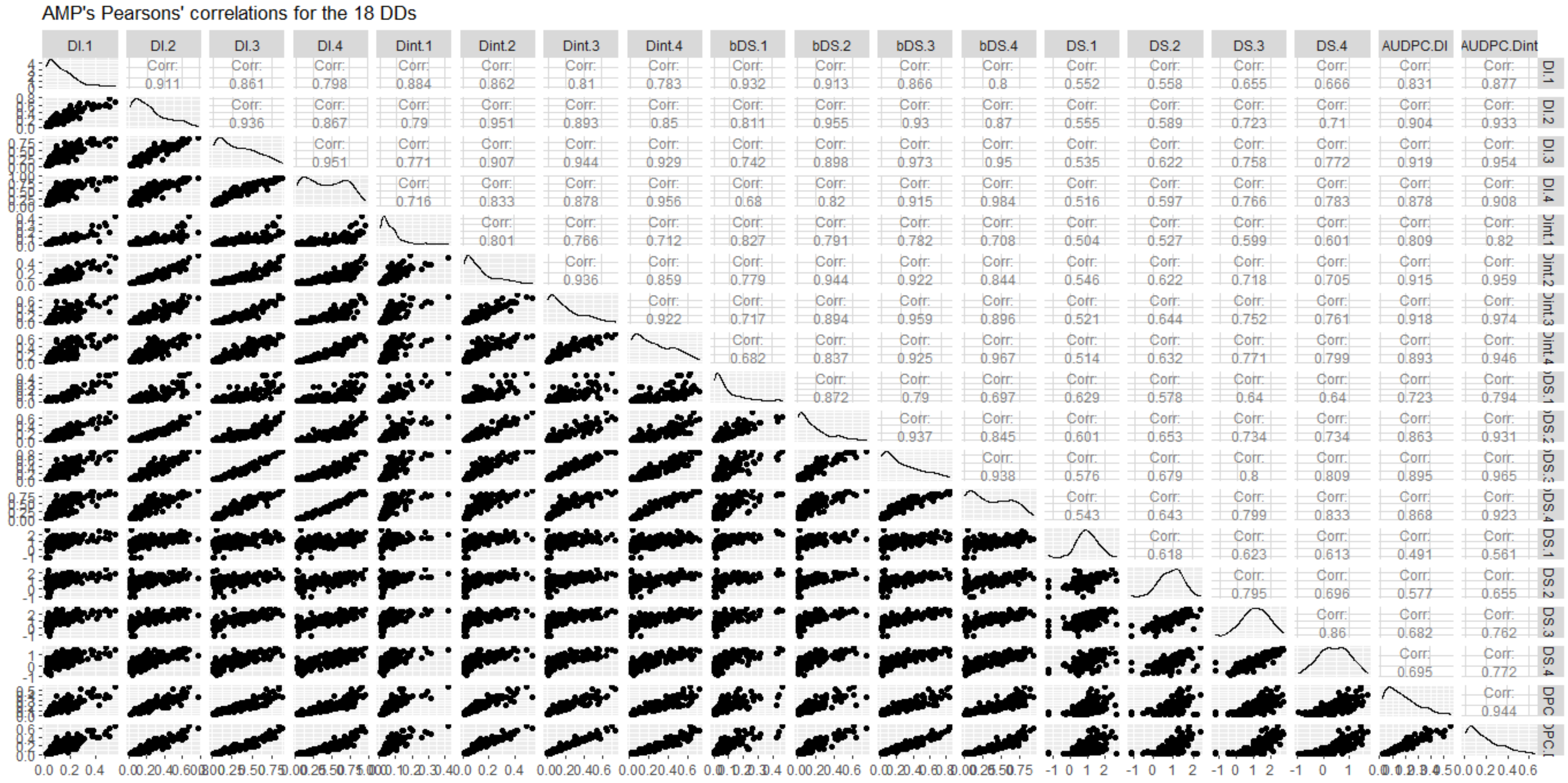

Supplementary Figure S3. Pearson's Correlations between IL's adjusted means for 18 DDs in the AMP. Below the diagonal: Scatter Plots of AMP-ILs' adjusted means for 18 DDs; Above the diagonal: Pearson's correlation coefficients between AMP-ILs' adjusted means for 18 DDs; Diagonal: Density plots showing the distribution of adjusted means frequencies.

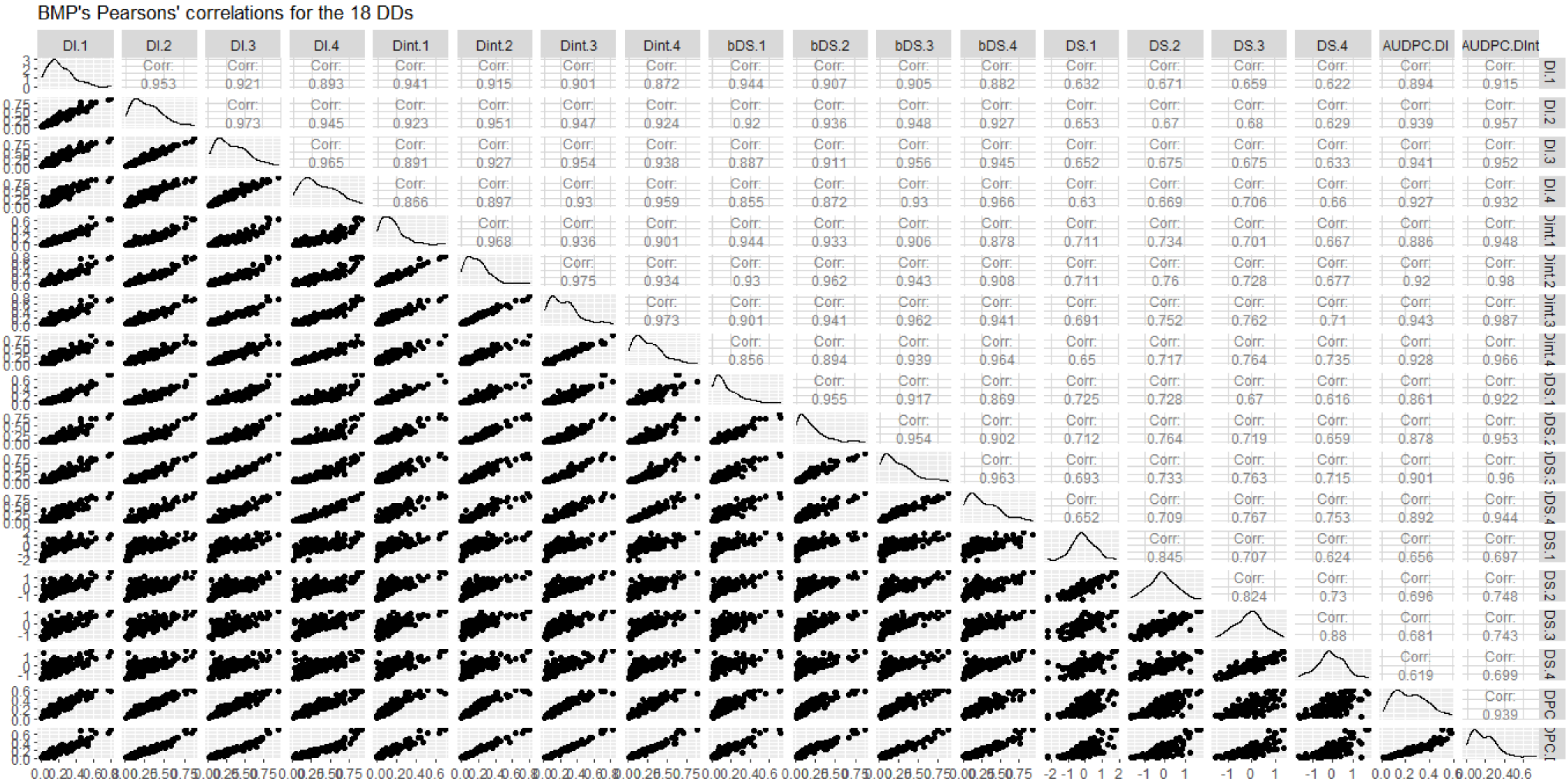

Supplementary Figure S4. P Pearson’s Correlations between RIL’s adjusted means for 18 DDs in the BMP. Below the diagonal: Scatter Plots of BMP-RILs’ adjusted means for 18 DDs; Above the diagonal: Pearson’s correlation coefficients between BMP-RILs’ adjusted means for 18 DDs; Diagonal: Density plots showing the distribution of adjusted means frequencies.

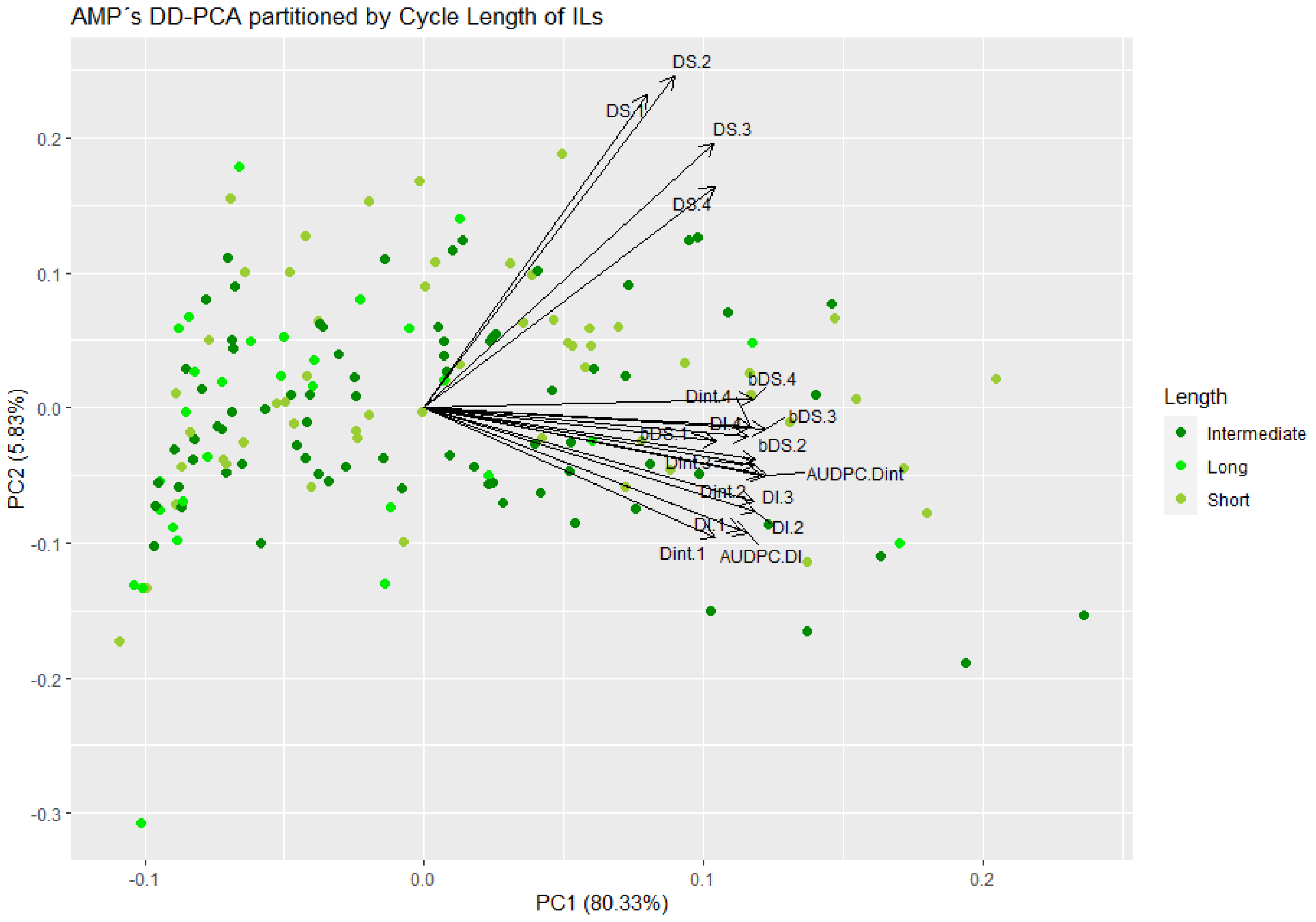

Supplementary Figure S5. Biplot of PCA of the AMP. Colors represent the Cycle Length of the 162 ILs evaluated for SVW-BSR.

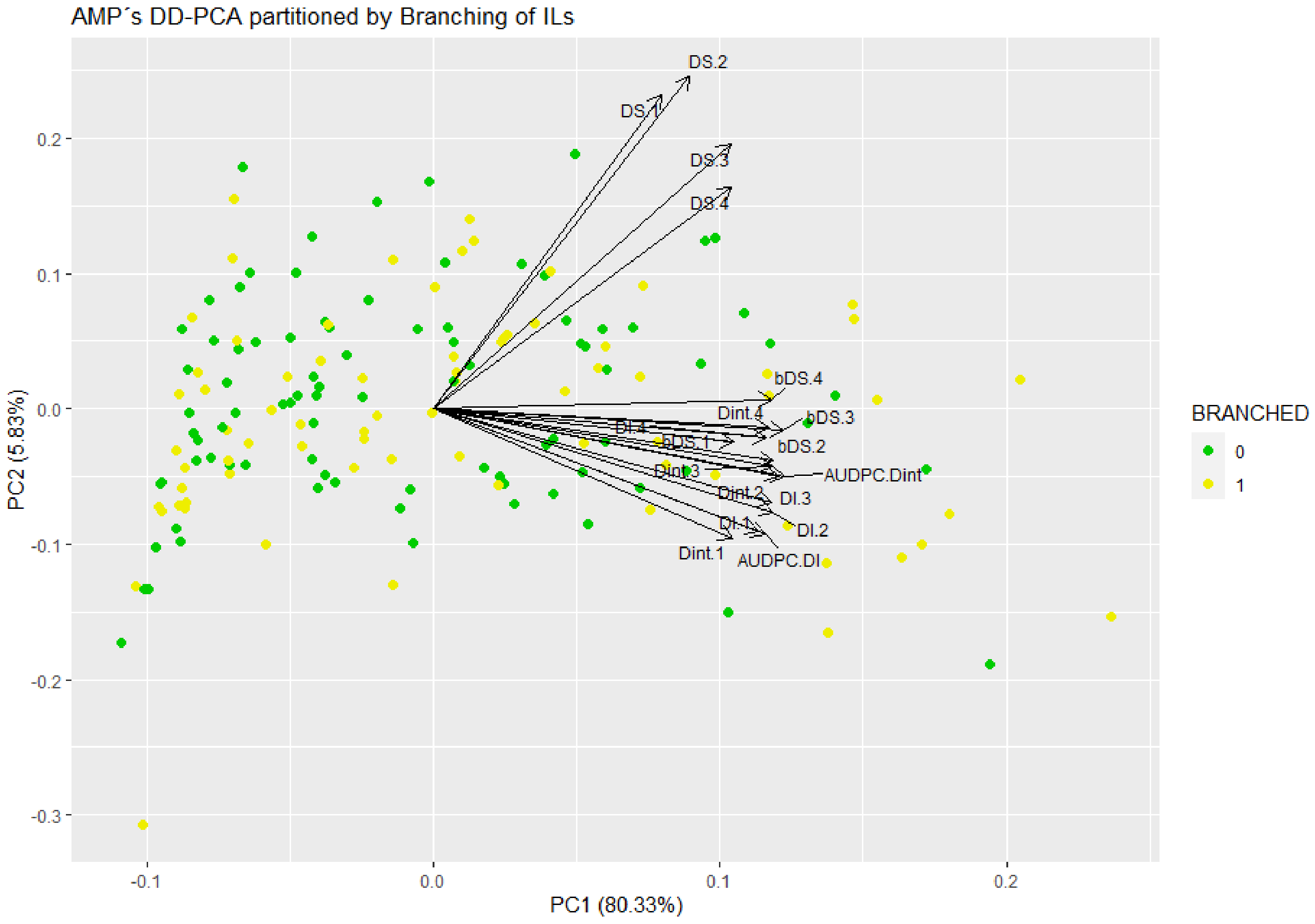

Supplementary Figure S6. Biplot of PCA of the AMP partitioned by branching status of the 162 ILs evaluated for SVW-BSR.

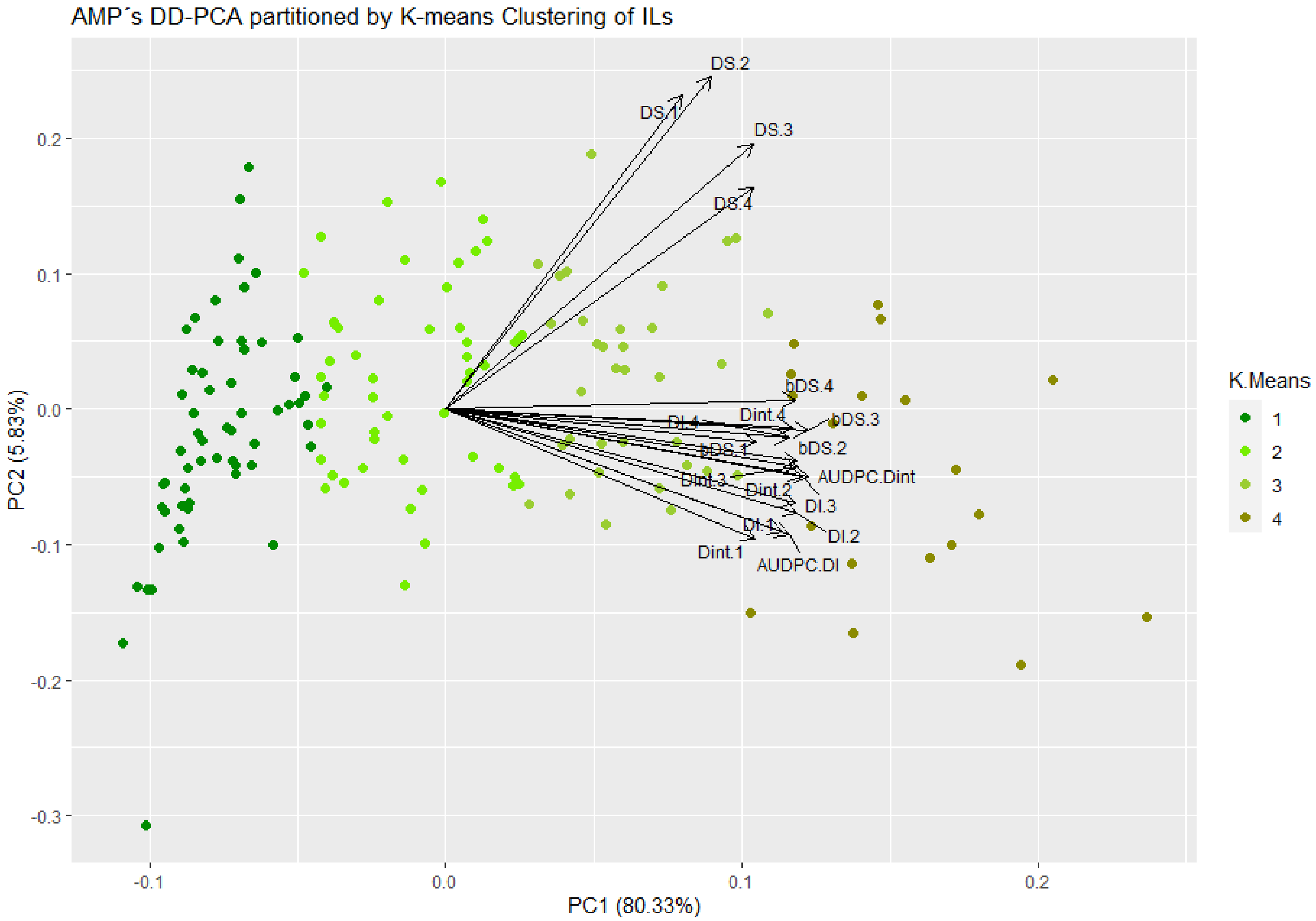

Supplementary Figure S7. Biplot of PCA of the AMP partitioned by k-means clustering method. Higher levels of SVW-BSR in clusters with lower numbers.

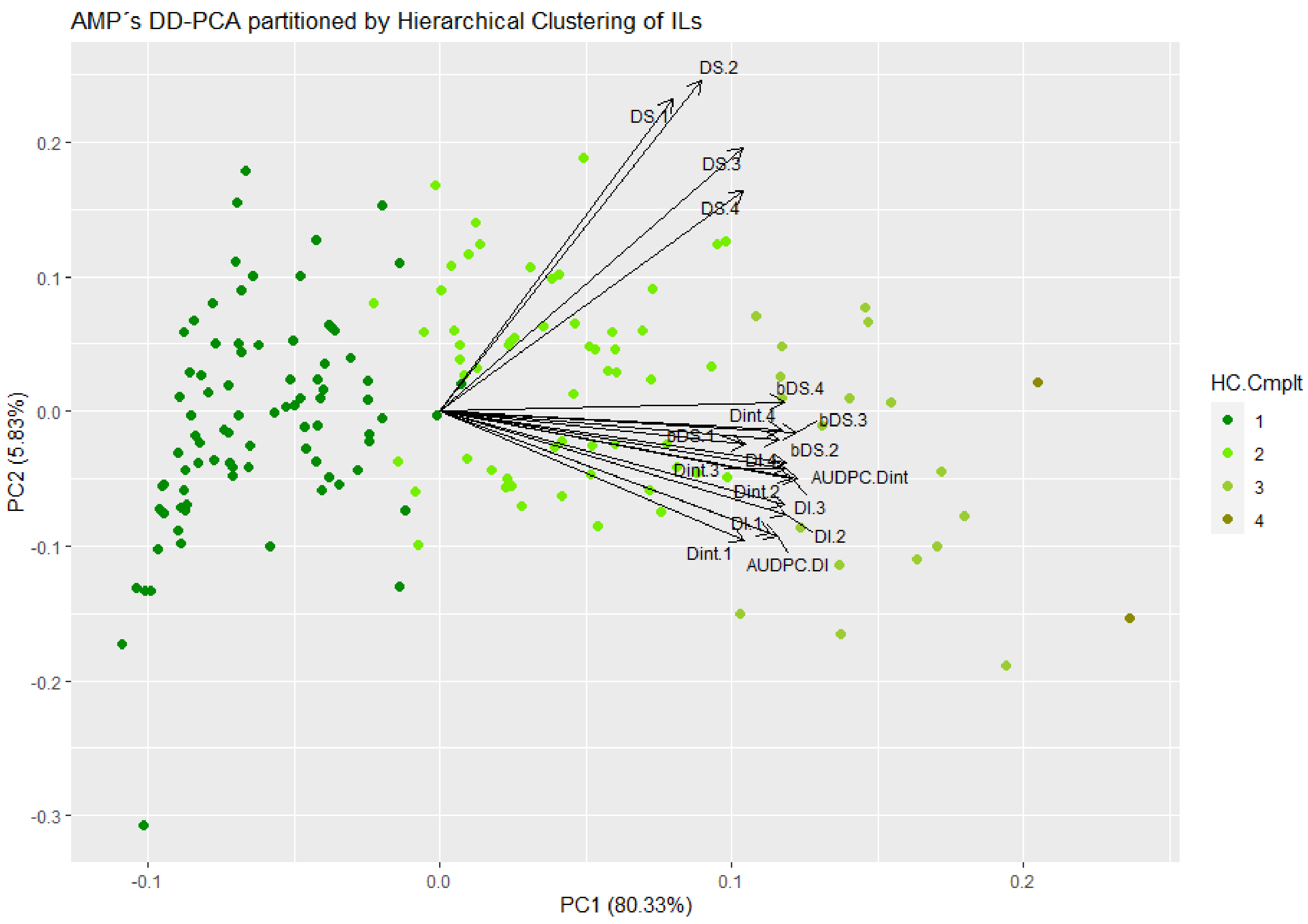

Supplementary Figure S8. Biplot of PCA of the AMP partitioned by hierarchical clustering method (Average). Higher levels of SVW-BSR in clusters with lower numbers.

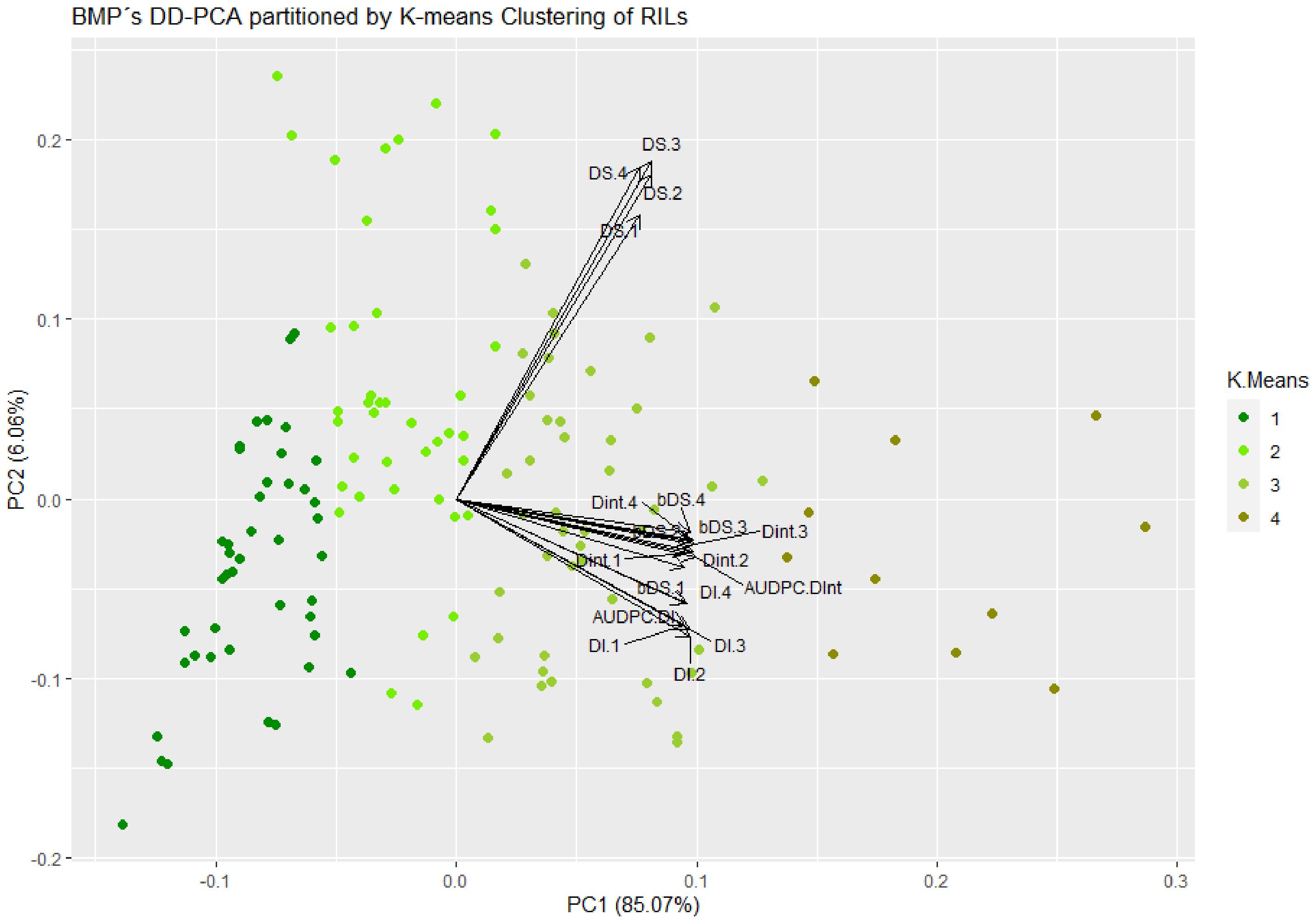

Supplementary Figure S9. Biplot of PCA of the BMP partitioned by k-means clustering method. Higher levels of SVW-BSR in clusters with lower numbers.

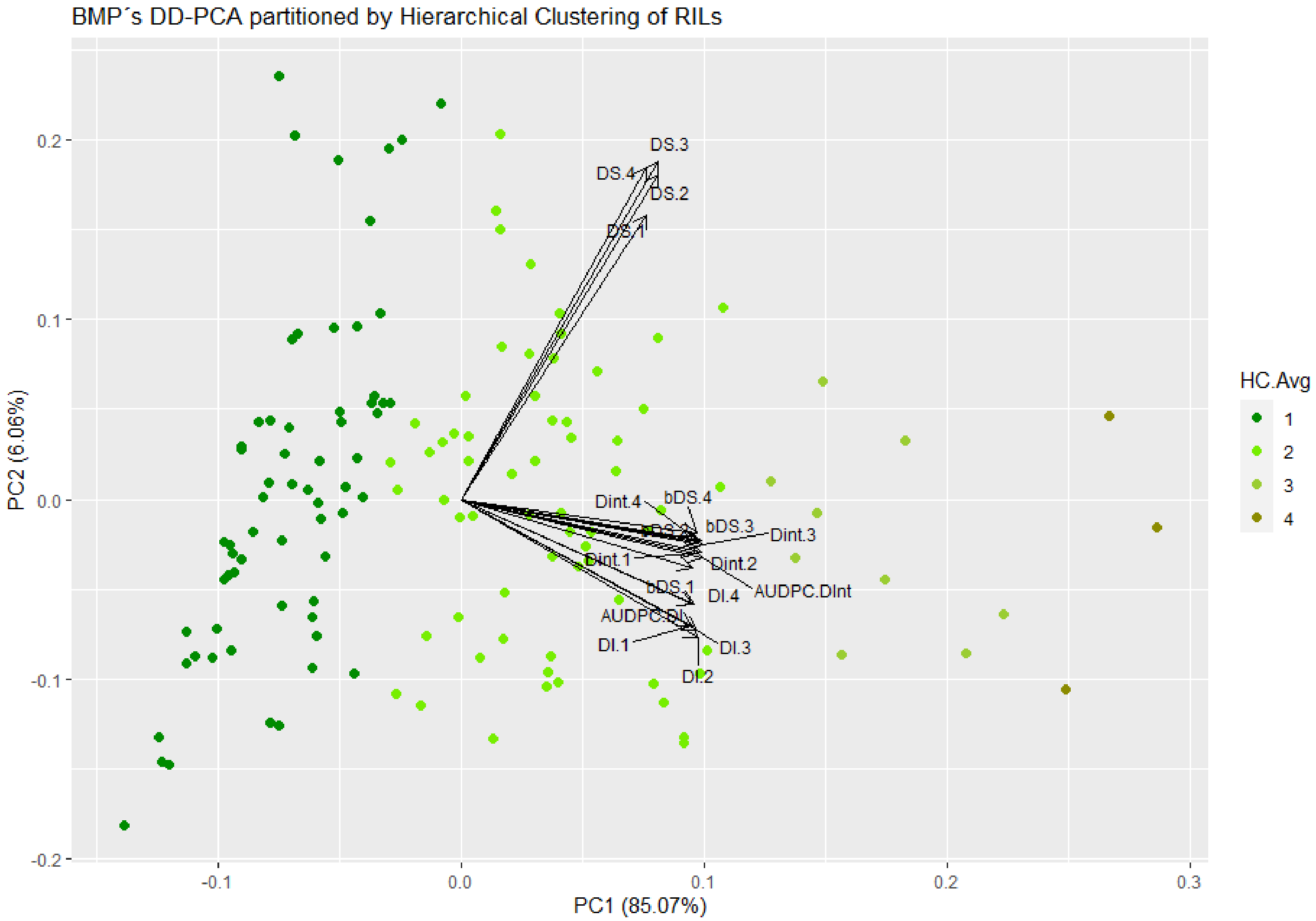

Supplementary Figure S10. Biplot of PCA of the BMP partitioned by hierarchical clustering method (Average). Higher levels of SVW-BSR in clusters with lower numbers.

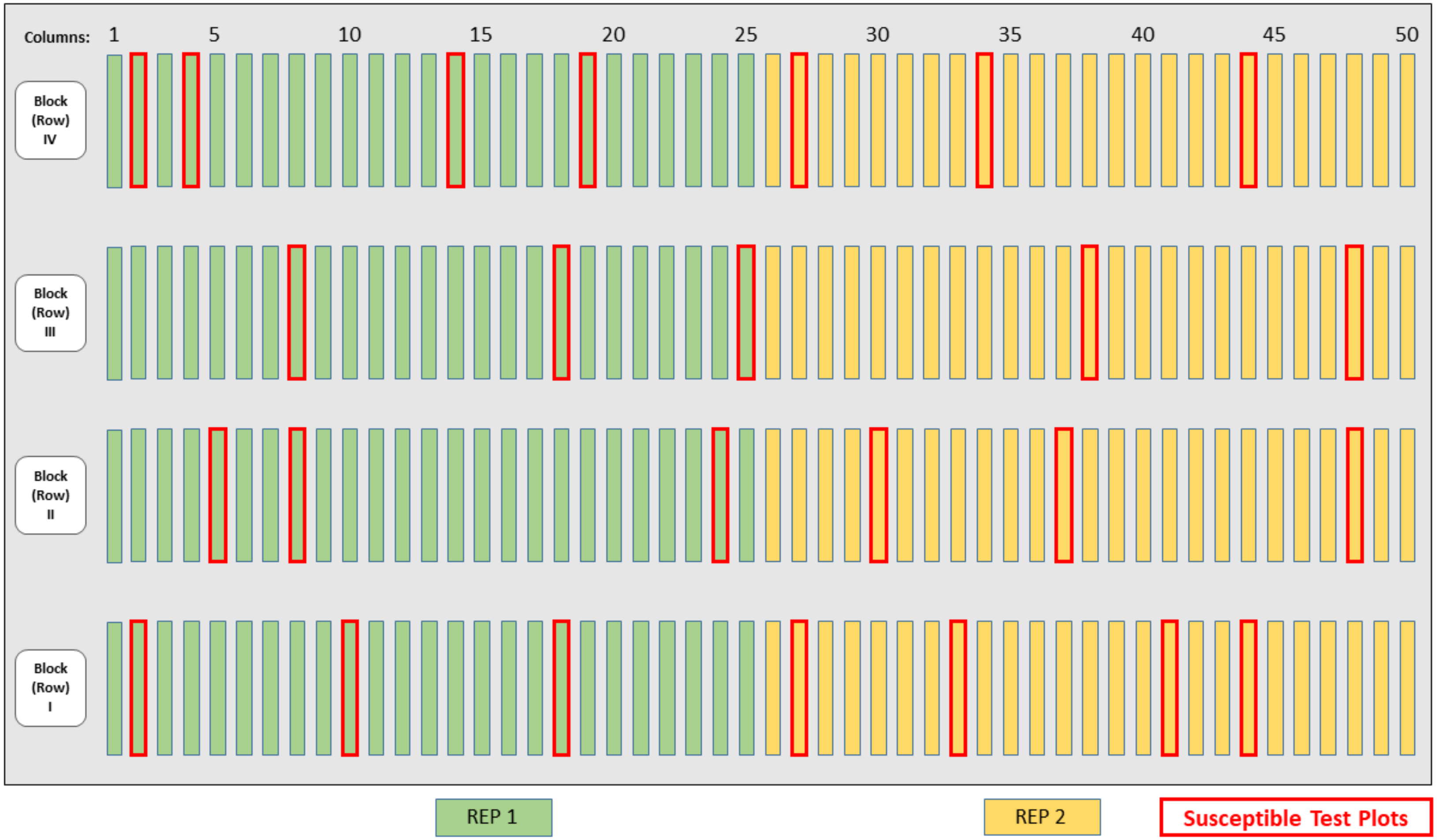

Supplementary Figure S11. Scheme of the Design of a regular Field Trial for SVW-resistance phenotyping. Vertical rectangles represent plots, Repetitions are denoted by color, Blocks (Rows for the Row-Column design) are defined by the group of parallel plots within a replication, Columns are conformed by plots placed over the same sowing line (numbered on top) and randomly distributed susceptible test plots are framed in red.

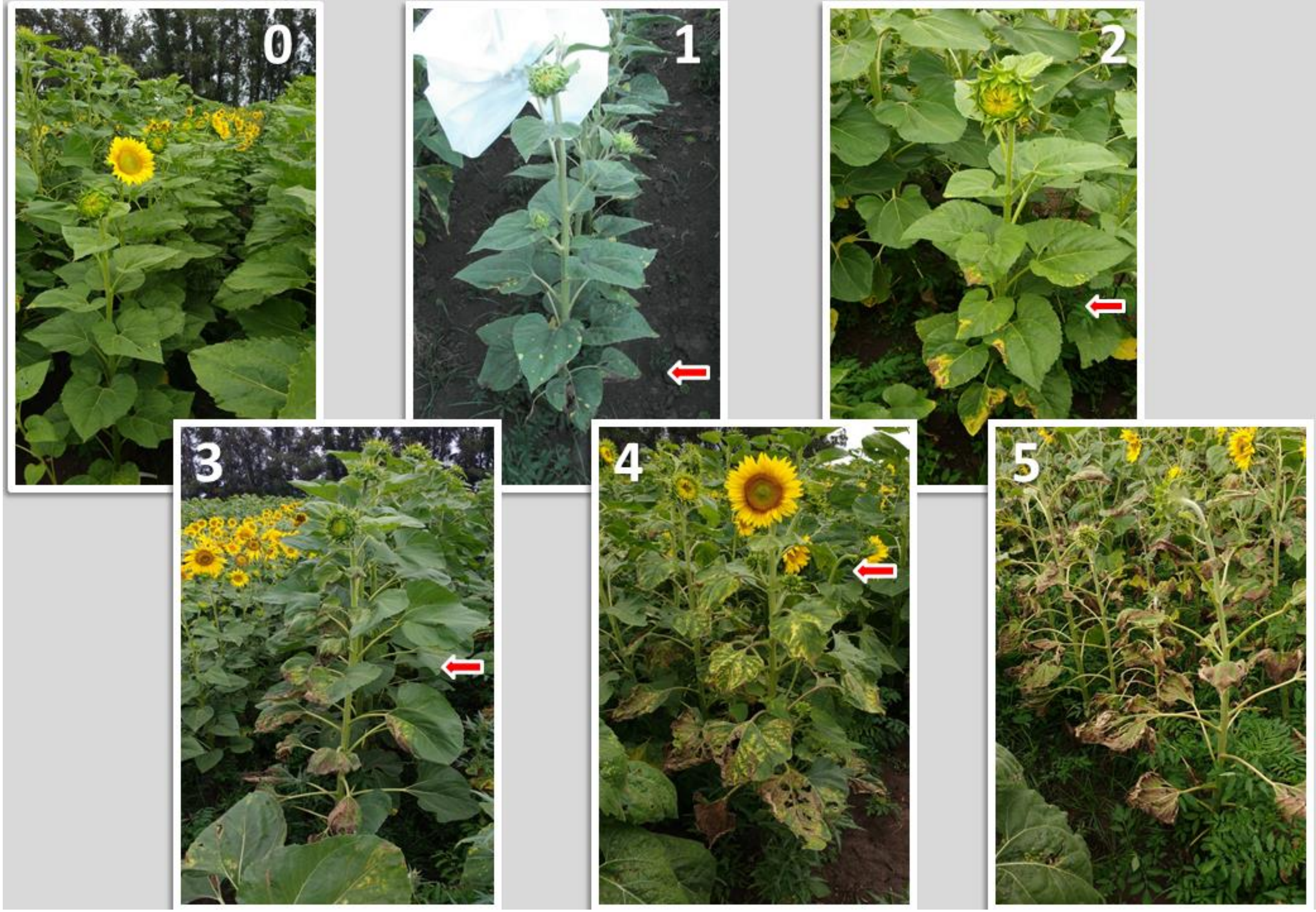

Supplementary Figure S12. SVW Disease Severity Scale. Ordinal scale of six levels from “0” for asymptomatic plants to “5” for totally wilted plants.
